# Aneuploidy-induced proteotoxic stress can be effectively tolerated without dosage compensation, genetic mutations, or stress responses

**DOI:** 10.1186/s12915-020-00852-x

**Published:** 2020-09-08

**Authors:** Katherine E. Larrimore, Natalia S. Barattin-Voynova, David W. Reid, Davis T. W. Ng

**Affiliations:** 1 Temasek Life Sciences Laboratory, Singapore, 117604 Singapore; 2 Current address: Institute of Medical Biology (IMB), Agency for Science, Technology and Research (A*STAR), Singapore, 138648 Singapore; 3 Duke-NUS Graduate Medical School, Singapore, 169857 Singapore; 4 Current address: Moderna Inc., Cambridge, MA 02139 USA; 5 Department of Biological Sciences, National University of Singapore, Singapore, 117543 Singapore

**Keywords:** Proteostasis, Aneuploidy, Protein homeostasis, Proteotoxic stress, Protein quality control

## Abstract

**Background:**

The protein homeostasis (proteostasis) network maintains balanced protein synthesis, folding, transport, and degradation within a cell. Failure to maintain proteostasis is associated with aging and disease, leading to concerted efforts to study how the network responds to various proteotoxic stresses. This is often accomplished using ectopic overexpression of well-characterized, model misfolded protein substrates. However, how cells tolerate large-scale, diverse burden to the proteostasis network is not understood. Aneuploidy, the state of imbalanced chromosome content, adversely affects the proteostasis network by dysregulating the expression of hundreds of proteins simultaneously. Using aneuploid haploid yeast cells as a model, we address whether cells can tolerate large-scale, diverse challenges to the proteostasis network.

**Results:**

Here we characterize several aneuploid *Saccharomyces cerevisiae* strains isolated from a collection of stable, randomly generated yeast aneuploid cells. These strains exhibit robust growth and resistance to multiple drugs which induce various forms of proteotoxic stress. Whole genome re-sequencing of the strains revealed this was not the result of genetic mutations, and transcriptome profiling combined with ribosome footprinting showed that genes are expressed and translated in accordance to chromosome copy number. In some strains, various facets of the proteostasis network are mildly upregulated without chronic activation of environmental stress response or heat shock response pathways. No severe defects were observed in the degradation of misfolded proteins, using model misfolded substrates of endoplasmic reticulum-associated degradation or cytosolic quality control pathways, and protein biosynthesis capacity was not impaired.

**Conclusions:**

We show that yeast strains of some karyotypes in the genetic background studied here can tolerate the large aneuploidy-associated burden to the proteostasis machinery without genetic changes, dosage compensation, or activation of canonical stress response pathways. We suggest that proteotoxic stress, while common, is not always an obligate consequence of aneuploidy, but rather certain karyotypes and genetic backgrounds may be able to tolerate the excess protein burden placed on the protein homeostasis machinery. This may help clarify how cancer cells are paradoxically both highly aneuploid and highly proliferative at the same time.

## Background

The protein homeostasis (proteostasis) network is a complex system that regulates the proteome by balancing protein synthesis and degradation. It can also detect imbalances such as increased flux of damaged or misfolded proteins and return the system to homeostasis by altering biosynthetic and degradative functions. Disruptions that cause such imbalances is collectively called “stress” and varying forms of stress to the protein quality control system can lead to human neurodegenerative diseases such as Alzheimer’s and Parkinson’s diseases [1]. One of the most extreme sources of stress to the proteostasis network is the aneuploid state which disrupts the normal regulation of protein quality control by altering expression of hundreds or thousands of genes simultaneously simply through gene copy number changes [2]. If left unchecked, excess protein molecules translated from the additional chromosomes unable to join partners to form complexes can misfold and form toxic species leading to fitness defects [3–6]. Similarly, increasing the levels of entire balanced protein complexes can also cause immediate and specific dysfunctions [7]. But the most basic problem in cases such as aneuploidy is the heavier burden on the protein synthetic system and the raw materials used to support it.

To study homeostasis maintenance in response to large and diverse burden on the proteostasis network, we chose to use the aneuploid state as a naturally occurring, extreme source of proteotoxic stress. Understanding if and how cells can tolerate such proteostasis challenges is relevant in the context of cancer which has the hallmarks of being both highly proliferative and aneuploid. Indeed, an astonishing ~ 90% of human solid tumors are aneuploid yet also exhibit uncontrollable proliferative capacities [8], suggesting the existence of mechanisms that can overcome the detrimental burden extreme stressors such as aneuploidy put on the proteostasis system. While cellular tolerance to extreme forms of proteotoxic stress undoubtedly involves a variety of different mechanisms depending on the particular genetic and environmental background of cells investigated [9–12], one potential area that has emerged as a therapeutic target involves protein degradation [13].

When faced with sudden, dramatically increased substrate loads, protein quality control pathways may become saturated and can lead to the activation of proteotoxic stress response pathways in order to endure the deleterious condition. While these responses, such as the heat shock response (HSR) or unfolded protein response (UPR), promote survival during temporarily stressful times, prolonged activation can be toxic in the long term [14]. Thus, it is unsurprising that the aneuploid state is generally deleterious for overall cellular health and viability [10, 15–17]. Indeed, aneuploid-sensitive strains in the W303 yeast background exhibit hallmarks of proteotoxic stress including accumulation of misfolded proteins and aggregates, severe growth defects, and sensitivity to drugs that place additional stress on the proteostasis network including protein translation, folding, trafficking, and turnover [11, 15, 18, 19]. Additionally, similar aneuploidy-associated proteostasis-related stresses and sensitivities to proteasome inhibitors are observed in mammalian cells [10, 16, 20, 21]. While many aneuploid cells may exhibit proteotoxic-related stress and proteasome inhibitor sensitivity, it has also been reported that some aneuploids with growth defects can acquire mutations that alter ubiquitin/proteasome system activities leading to improved fitness [11, 22]. However, the correct genetic changes can take many generations to accumulate and mutations are not observed in all tolerant aneuploid strains indicating additional mechanisms of aneuploidy tolerance exist.

In this study, we sought to understand if aneuploid cells of specific karyotypes in a model organism exhibit physiologic tolerance to chronic, large-scale, diverse proteostasis burden. To address this, we used robust haploid aneuploid strains from a collection of stable, randomly generated yeast aneuploid cells [17]. To investigate how cells handle stepwise, increasing protein load brought on by aneuploidy while also introducing the least amount of karyotypic heterogeneity between strains, we randomly selected four haploid, stable, isogenic aneuploid yeast strains which have incremental increasing excess chromosomal content (Disome (D) D2, D1/2, D1/2/8, and D1/2/8/11) as well as strains without a duplicated chromosome II (D1/8 and D13). A previous systematic yeast aneuploid study reported that strains disomic for single chromosomes exhibited reductions in fitness from minimal to severe, according to the duplicated chromosome [15]. The fitness loss was dramatic and additive for different strains disomic for just two chromosomes, reflecting increasing stress with greater chromosome numbers. In contrast, the strains analyzed in the current study which have increasing number of duplicated genes did not show corresponding increased severity of growth defects. This points to a relatively high degree of aneuploidy tolerance for these karyotypes, perhaps as a result of the method of random meiotic segregation and selection of stable, viable progeny used to generate these aneuploid cells. Genome sequencing of multiple strains confirmed that their tolerance to aneuploidy is due to intrinsic mechanisms and not genetic mutation. Global expression profiling experiments revealed karyotype-specific expression signatures such as coordinate upregulation of protein biosynthetic and quality control pathways that does not include constitutive activation of known stress pathways. Direct biochemical analyses demonstrated no severe defects of the protein quality control system, indicating that the proteostasis pathways are functionally sufficient to handle the extreme protein load in the aneuploid strains.

## Results

### Robust growth and enhanced stress tolerance indicate physiologic tolerance to aneuploidy-associated proteotoxic stress

**Fig. 1:**
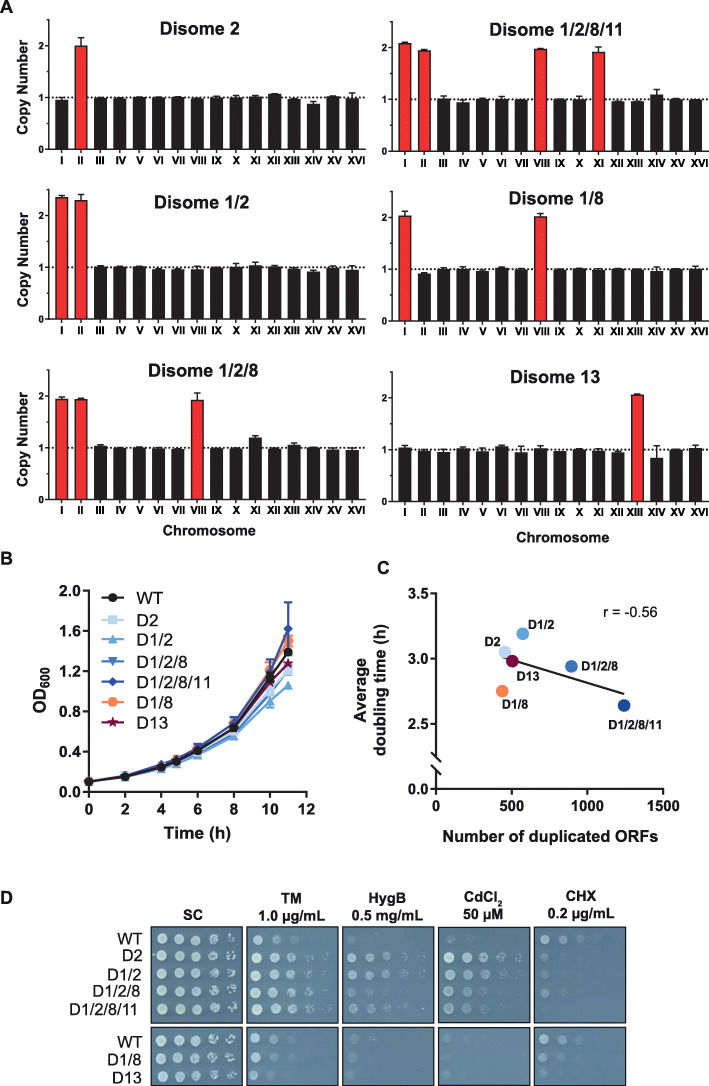
Robust growth and enhanced stress tolerance indicate physiologic tolerance to aneuploidy. **a** Karyotypes of six aneuploid strains were determined by qPCR. Given chromosome copy numbers are quantified relative to their euploid (wild type, WT) levels and duplicated chromosomes are highlighted in red. Error bars indicate SD between the left chromosome arm and right arm of one biological replicate (see Methods). **b** Growth rates of yeast strains (mean of 3 biological replicates ± SEM) in liquid synthetic complete (SC) media at 25 °C. Growth rate was monitored over time by measuring optical density at the times indicated. **c** Average doubling times of indicated yeast strains plotted against the number of additional aneuploidy-associated duplicated open reading frames. **d** Proliferation of WT and aneuploid strains grown on SC the presence of tunicamycin (TM, 1 μg/mL), hygromycin B (HygB, 0.5 mg/mL), cadmium chloride (CdCl_2_, 50 μM), or cycloheximide (CHX, 0.2 μg/mL). Fivefold serial dilutions of indicated strains were spotted on SC medium with or without indicated treatments. Three independent experiments were performed, all yielding consistent observations. Representative plate scans are shown

To understand the molecular events enabling tolerance to a range of simple to complex aneuploid states, we took advantage of an established model in budding yeast [17]. We chose strains originally generated through meiosis of odd ploidy (3N or 5N) homozygotes from the S288C strain background [17]. Because karyotypes resulted from a stochastic process, the collection seemed to hold promise to identify a subset of robust, aneuploid-tolerant strains. From it, six new aneuploid strains (haploid base plus duplicated whole chromosomes) NVY1, NVY2, NVY3, KLY193, KLY194, and KLY196 (Additional file 1: Table S1) were isolated. The isogenic haploid euploid parental strain (RLY2626) will be referred to as “wild type” or WT. Karyotypes were determined by the previously described qPCR-based assay (Fig. 1a, Additional file 2: Table S2) [17]. For simplicity, we will refer to NVY1, NVY2, NVY3, KLY193, KLY194, and KLY196 as D2, D1/2, D1/2/8, D1/2/8/11, D1/8, and D13, respectively, with “D” signifying “Disome” followed by the duplicated chromosome number(s).

The number of open reading frames (taken from Saccharomyces Genome Database) encoded on the excess chromosomes in the aneuploid strains ranges from 438 (D1/8), 456 (D2), 505 (D13), 573 (D1/2), 894 (D1/2/8), and 1242 (D1/2/8/11). Despite increasing levels of excess DNA content, all six strains grow similarly to the euploid parental strain (Fig. 1b). There was no positive correlation observed between growth doubling time and extra DNA content (Pearson’s *r* = − 0.56, Fig. 1c). Whole genome sequencing was performed for strains WT, D2, D1/2, D1/2/8, and D13 which confirmed karyotyping results and verified the absence of large segmental chromosome abnormalities (e.g., loss or gain of partial chromosomes) (see Additional file 3: Fig. S1, Methods, whole genome sequence data are available on NCBI’s SRA database under BioProject accession number PRJNA590310).

To assess whether aneuploidy alters proteotoxic stress sensitivity, the cells were grown in the presence of various compounds that elicit cell stress. Four of the strains, which all carry in common a duplicated chromosome II, displayed greater resistance to the proteotoxic compounds tunicamycin, hygromycin B, and cadmium chloride, whereas strains D1/8 and D13 were more sensitive to these compounds (Fig. 1d). Disome 2 strains engineered to lack the excess gene copies of PCA1, a cadmium transporter [23], or PDR3, a transcriptional activator of the pleiotropic drug resistance network [24], retained enhanced resistance to the drugs (Additional file 4: Fig. S2A, S2B). Although the heavy metal cadmium can cause a range of biochemical dysfunctions, tunicamycin and hygromycin B specifically promote protein misfolding by inhibiting N-linked glycosylation and reducing translational fidelity, respectively. To confirm the activity of tunicamycin, we monitored the glycosylation of the well-studied endogenous glycoprotein carboxypeptidase Y (CPY) [25]. Indeed, underglycosylated forms of CPY are observed in all tested strains treated with tunicamycin, demonstrating efficacy of the drug (Additional file 4: Fig. S2C). Against the general translation inhibitor cycloheximide (CHX), all aneuploid strains displayed strong sensitivity, presumably due to their greater need for biosynthetic capacity (Fig. 1d) [15, 17]. Together, these results indicate that increased chromosomal burden in aneuploid-tolerant strains is not correspondingly increasingly detrimental to cellular growth or general proteostasis function.

### Aneuploid cells upregulate broad facets of the protein homeostasis network without chronic activation of stress pathways

**Fig. 2:**
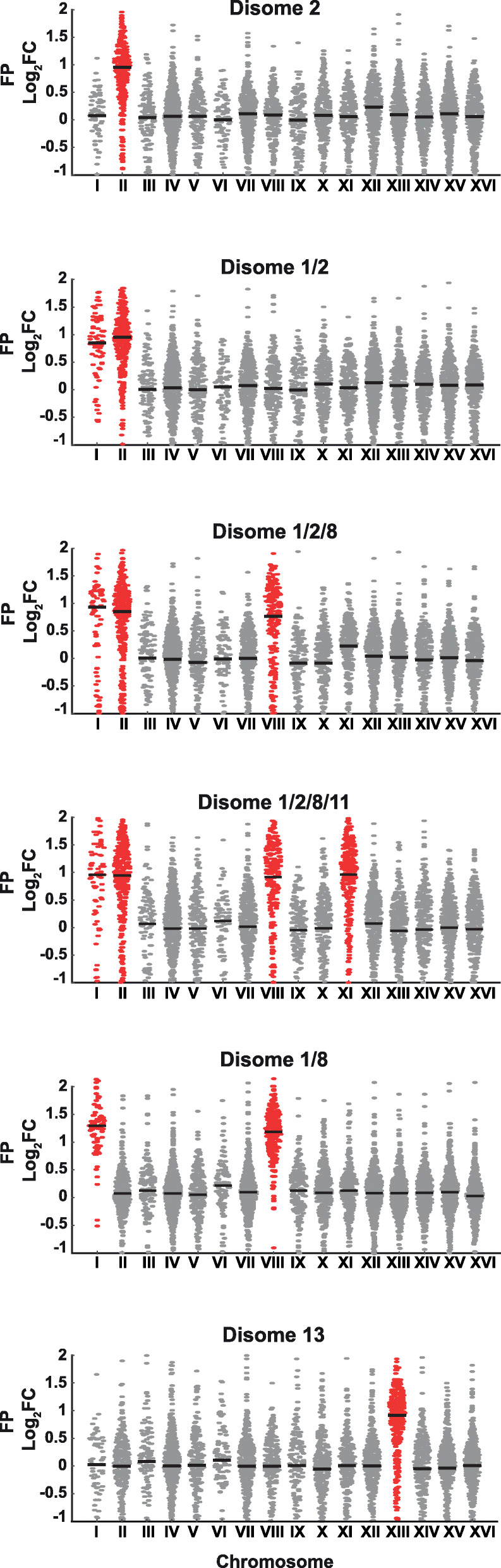
Genes encoded on duplicated chromosomes undergo efficient translation in tolerant aneuploid yeast strains. Relative levels of the ribosome-protected footprint (FP) fragments in aneuploid strains compared to the wild type (WT) cells were analyzed by ribosome profiling. Each gene is shown as a circle plotted at its log2 fold change (FC) of FP in the aneuploid strains relative to the WT control with median indicated with a black bar and duplicated chromosomes highlighted in red

To determine transcriptional and translational changes in the aneuploid strains compared to the euploid control, we performed transcriptional profiling (RNA-Seq) combined with ribosome footprinting (deep sequencing of ribosome-protected mRNA fragments) on the euploid and aneuploid strains D2, D1/2, D1/2/8, D1/2/8/11, D1/8, D13, and a D13 strain that has WT sequences in CAK1 and SEC24 (KLY195) [26]. The development of global ribosome profiling technologies by the Weissman group revealed that translation control is widespread so applying this technology provides a clearer regulatory map than transcriptional profiling alone [26, 27]. Relative levels of ribosome-protected footprint (FP) fragments (Fig. 2) and mRNA abundance and ribosome density (Additional file 5: Fig. S3) in aneuploid strains were compared to euploid control. The majority of genes are transcribed and translated in accordance to chromosome copy numbers, and thus, large-scale dosage compensation is not a mechanism of aneuploidy tolerance in these strains.

**Fig. 3:**
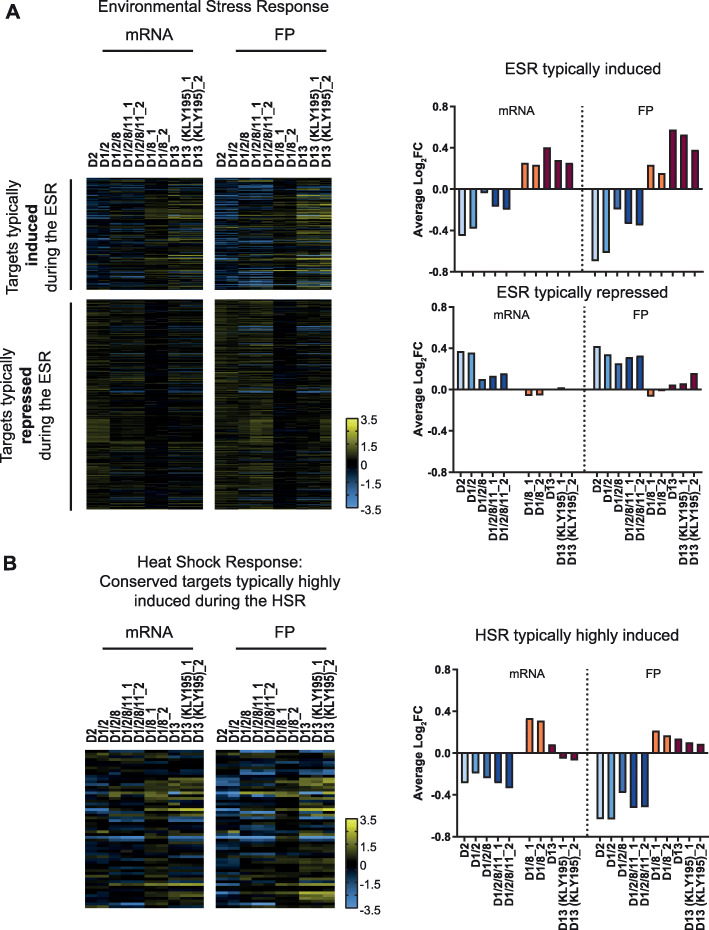
Tolerant aneuploid strains do not induce a common proteotoxic stress response signature. Genes involved in stress responses were identified from mRNA-seq (mRNA) and ribosome profiling footprint (FP) datasets. Only genes residing on nonduplicated chromosomes and for which complete datasets were available for all strains were included in the heatmap analysis. The columns labeled with _1 and _2 indicate respective biological replicates of a given strain. The average log2 fold change (FC) mRNA and FP expression levels in the aneuploid strains relative to the WT of all nonduplicated genes involved in a given response were calculated (bar graphs). **a** Genes involved in the environmental stress response (ESR) (gene list from [28]) were separated in two groups: those induced during the canonical ESR (top) and those repressed (bottom). **b** Genes induced during the heat shock response (HSR) that are strongly induced across multiple species in response to heat (gene list from [29]). The following figure supplements are available for Fig. 3: Additional file 6: Fig. S4, Additional file 7: Fig. S5 and Additional file 8: Table S3

Next, we sought to understand if the cells were utilizing stress response pathways to enhance protein homeostasis capacity. We analyzed the expression pattern of genes involved in various stress response pathways and did not observe broad activation of the environmental stress response (ESR) [28] or the heat shock response (HSR) [29] in strains D2, D1/2, D1/2/8, and D1/2/8/11 (Fig. 3a, b). Strain D13, and to a modest extent D1/8, did exhibit a gene expression signature similar to that of the ESR (Fig. 3a). Direct biochemical analysis of the unfolded protein response (UPR) demonstrated that the major proteostasis regulatory pathway of the endomembrane system is not active (Additional file 6: Fig. S4A-C) [25]. Indeed, the UPR signaling protein, Ire1, can be deleted in tested strains causing only minor reduction in the hyper-resistance to tunicamycin indicating that the observed drug resistance uses a mechanism independent of the UPR (Additional file 6: Fig. S4D). While these data do not exclude the activation of stress pathways during the initial transition from euploidy to aneuploidy, they show that stress pathways are not chronically activated in some aneuploid strains, specifically those with chromosome II in excess.

Previous studies reported that aneuploidy can increase reactive oxygen species and induce the oxidative stress response (OSR) [30]. OSR-regulated genes [31] were mildly activated across all strains at the mRNA level, but not at the FP level in strains D2 and D1/2 (Additional file 7: Fig. S5A). To characterize the pathway responsible for the activation of redox-related genes, we further analyzed the mRNA and FP expression of genes typically activated in response to Yap1 and hydrogen peroxide (H_2_O_2_) [32, 33]. At the FP level, there was a broad upregulation of most YAP1-inducible targets (Additional file 7: Fig. S5B). An H_2_O_2_-dependent stress response appeared most highly induced in strains with a duplicated chromosome XIII (Additional file 7: Fig. S5C-D). The source data used for generating Fig. 3, including the gene names that are plotted, are provided (Additional file 8: Table S3). In the remaining aneuploid strains, specific genes including certain antioxidant scavenging genes, genes involved in protein degradation, chaperones, and gene involved in translation machinery were upregulated.

**Fig. 4:**
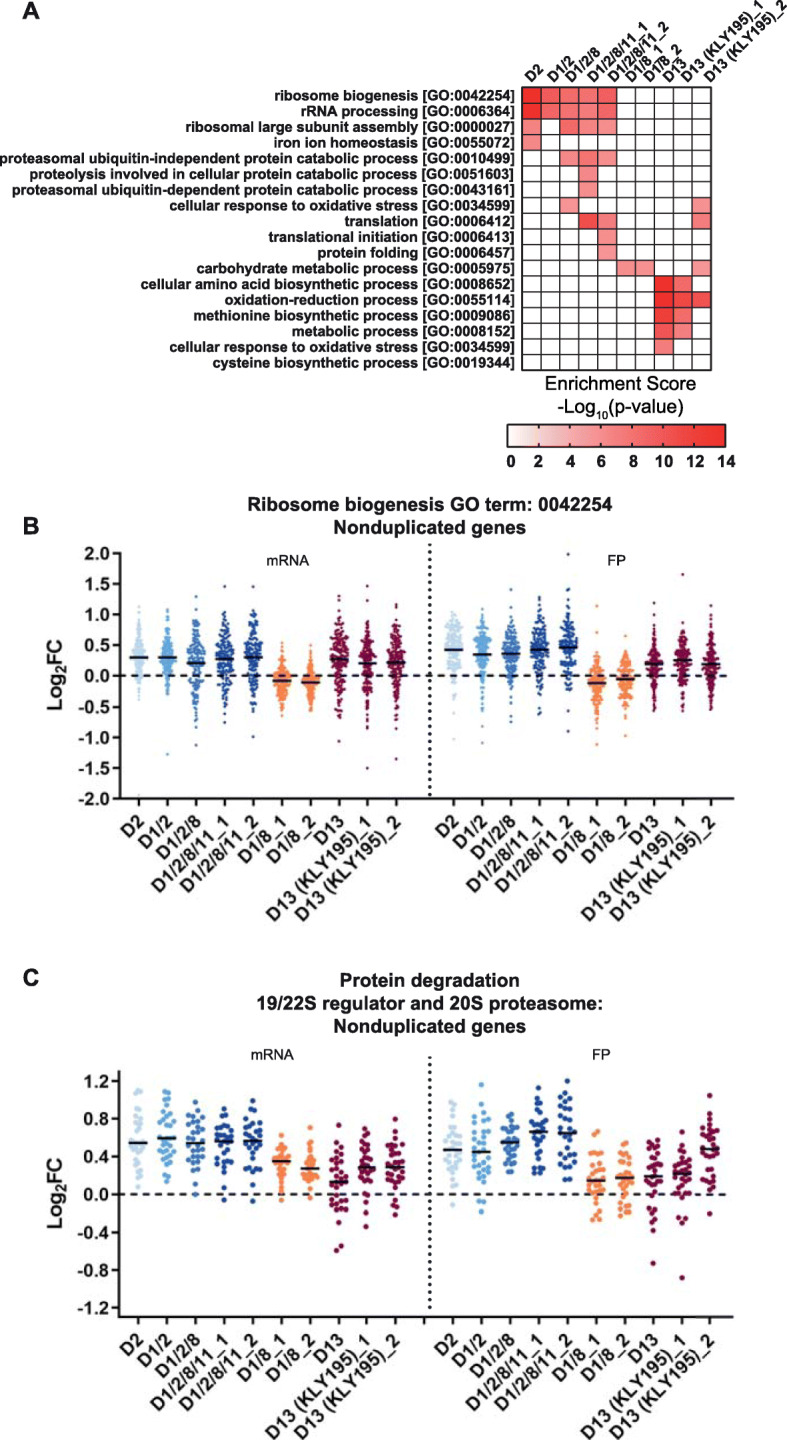
Facets of the protein homeostasis network are upregulated in multiple aneuploid strains. **a** GO term enrichment analysis with Bonferroni correction applied to genes residing on nonduplicated chromosomes that had at least 1.5-fold (0.585 log2 fold change) increase in ribosome-protected footprint (FP) levels relative to wild type (WT) for each strain. The columns labeled with _1 and _2 indicate respective biological replicates of a given strain. *p* values were calculated from hypergeometric tests following enrichment of GO terms relating to GO Biological Process Classification obtained at http://funspec.med.utoronto.ca/. **b** Relative mRNA and FP levels in aneuploid strains compared to the WT cells of individual nonduplicated gene members belonging to the ribosome biogenesis GO term annotation 0042254. Each gene is represented by a dot with a black bar indicating the mean. **c** Relative mRNA and FP levels in aneuploid strains compared to the WT cells of individual nonduplicated gene members belonging to the 19/22S proteasome regulator and 20S proteasome complex. Each gene is represented by a dot with a black bar indicating the mean. The following figure supplements are available for Fig. 4: Additional file 9: Fig. S6, Additional file 10: Fig. S7 and Additional file 11: Table S4

To explore common regulatory changes using an unbiased approach, GO term enrichment was conducted on nonduplicated genes differentially upregulated (Fig. 4) or downregulated (Additional file 9: Fig. S6) by at least 1.5-fold relative to the euploid control. The number of genes residing on nonduplicated chromosome that were upregulated at the FP level was 568 (D2), 506 (D1/2), 421 (D1/2/8), 577 and 642 (D1/2/8/11 biological replicates), 272 and 246 (D1/8 biological replicates), 611 (D13), and 549 and 623 (D13 KLY195 biological replicates). No GO term was commonly found to be significantly enriched across all aneuploid strains from the upregulated (Fig. 4a) or downregulated genes (Additional file 9: Fig. S6) indicating lack of a common stress response across all strains. Strains D1/2/8, D1/2/8/11, and D13 downregulated genes showed enrichment in various transport and metabolic processes, while no enrichment in downregulated genes (Additional file 9: Fig. S6) was found present specifically in only strains with chromosome II duplicated. Strain D13 was enriched in its upregulated FP genes for “oxidation-reduction process” which is consistent with the observed heightened OSR. Interestingly, strains disomic for chromosome II upregulate genes covering various facets of the protein homeostasis network. For D2, D1/2, D1/2/8, and D1/2/8/11, these included genes involved in ribosome biogenesis and rRNA processing. Expression levels of ribosome biogenesis-related genes increased approximately 30% at both the mRNA and FP levels (Fig. 4b). This contrasts the strong downregulation of ribosome-associated genes observed in slow-growing aneuploid strains that exhibit an ESR-like stress signature [15, 28]. Proteosome-associated protein degradation is enriched in strains D1/2/8 and D1/2/8/11. By querying proteasome-related gene expression levels, these genes were found coordinately upregulated across all strains (although more moderately in D1/8 and D13) at both mRNA and FP levels (Fig. 4c). Finally, the upregulated genes in strain D1/2/8/11 were also enriched in other factors involved in protein homeostasis processes including protein translation and protein folding (Fig. 4a). This suggests a rewiring of the proteostasis network without induction of proteostasis-related stress responses in some aneuploid strains.

We next asked what categories of genes are differentially regulated commonly across the four strains D2, D1/2, D1/2/8, and D1/2/8/11, as these strains indicated adjustments to the proteostasis network without inducing proteotoxic stress responses. Using a less stringent, arbitrary cutoff of 30% (0.38 log_2_ fold change) increase in FP over euploid, 251 upregulated genes (not present on chromosome 2) were identified in common across all four strains that have chromosome II duplicated (Table 1). Based on the descriptions of the biological processes for each gene as described in the Saccharomyces Genome Database (SGD), these were manually curated into twelve functional categories to overcome limitations of algorithmic-based GO term analysis which may mischaracterize genes using inaccurate GO term associations (Table 1).

**Table 1:** Genes upregulated at the ribosomal footprint level by at least 30% in Disomic 2 strains

**Ribosome homeostasis**	*ACL4*, *ARB1*, *ARX1*, *BMT5*, *BRX1*, *CBF5*, *DBP10*, *DBP7*, *DBP8*, *DHR2*, *DRS1*, *ECM1*, *EMG1*, *ESF1*, *GAR1*, *GRC3*, *HAS1*, *HPM1, IMP3*, *IPI3, JIP5*, *KRR1*, *LOC1*, *LSG1*, *MAK11*, *MAK16*, *MRT4*, *NIP7*, *NOP12*, *NOP2*, *NOP4*, *NSR1*, *POP5*, *PUF4*, *PWP1*, *RIX1, RIX7*, *RLP24*, *RMT2*, *RPF2*, *RPL15A*, *RPL1A*, *RPL1B*, *RPL24A*, *RPL24B*, *RPL3*, *RPL8B*, *RPL9A*, *RPL9B*, *RPS22A*, *RPS5*, *RPS8B, RRB1*, *RRP17*, *SAC3*, *SFM1*, *SPB1*, *SRP40*, *STM1*, *TMA19*, *UTP11*, *UTP14*, *UTP23*, *UTP30*, *UTP5*, *UTP9*
**Protein degradation/quality control**	*CDC26*, *CIC1*, *CUZ1*, *ECM29*, *NAS6*, *PRE1*, *PRE10*, *PRE2*, *PRE4*, *PRE9*, *RPN1*, *RPN4*, *RPN5*, *RPN8*, *RPT3*, *RPT4*, *RPT5*, *SCL1*, *SMT3*, *TMC1*
**Protein folding/chaperones**	*CCT4*, *CCT7*, *HCH1*, *HSP10*, *PPT1*, *SSA2*, *SSZ1*
**Trafficking**	*ARF1*, *GET3*, *HOT13*, *HSV2*, *MLP1*, *MYO4*, *NTF2*, *NUP100*, *NUP49*, *PEX4*, *RET3*, *SEC22*, *SEC72*, *SRM1*, *SRP21*, *TOM7*, *YKT6*, *YPT31*
**Metabolism**	*AAT1*, *ACS1*, *ALD6*, *ARO10*, *ASP1*, *CAB5*, *CAR1*, *CAT5*, *COX7*, *DFR1*, *DPH2*, *ECT1*, *ERG13*, *ERG9*, *FAU1*, *GEP4*, *GIP4*, *GUA1*, *GUK1*, *MRI1*, *NPY1*, *PDC1*, *PDC5*, *PRS1*, *PRS3*, *PRS5*, *SAH1*, *SES1*, *SFA1*, *SOL3*, *SPE3*, *SUT2*, *TDA9*, *URA5*, *YHR020W*, *YJU3*
**Transcription, translation**	*CDC33*, *DEF1*, *GCD1*, *HYP2*, *NET1*, *PDP3*, *RPA34*, *RPA49*, *RPB7*, *RPB8*, *RPC31*, *RPC40*, *SLF1*, *SUP35*, *TEF1*, *TFA1*, *TIF2*, *TIF5*, *TOA1*, *ZPR1*
**DNA and RNA processing**	*CWC2*, *GCD10*, *HRP1*, *LHP1*, *LSM12*, *LSM5*, *NAM8*, *PAB1*, *PAN3*, *PRP16*, *PRP19*, *PRP8*, *RRP42*, *RRP43*, *RSE1*, *SEN34*, *SET1*, *SNU23*, *SUB2*, *SWD1*, *SWM2*, *TAD2*, *TRM11*, *TRM12*, *TRM2*, *TRM8*
**Chromosome organization/DNA replication and repair**	*AHC2*, *CBF2*, *DSN1*, *FPR4*, *GSP1*, *MAG1*, *NSE1*, *PAA1*, *POL31*, *RSC4*, *RTT107*, *SGS1*, *TAH11*, *TRA1*
**Cell cycle/signaling**	*BUD2*, *CCR4*, *CKA2*, *CLG1*, *CLN3*, *DMA1*, *HST2*, *KIN3*, *KSP1*, *LTE1*, *MAD1*, *NAT5*, *SCH9*, *TOS3*, *TPD3*
**Redox homeostasis**	*AHP1*, *GRX3*, *HMX1*, *LOT6*, *MRS4*, *TAH18*, *TSA1*, *YAP1*
**Others**	*EMW1*, *LRE1*, *OCA5*, *PTP2*, *YPD1*
**Putative/unknown function**	*AIM29*, *ANR2*, *ECL1*, *GDS1*, *NRP1*, *PMU1*, *RRT14*, *UBP5*, *VPS63*, *YCR043C*, *YDR222W*, *YEL068C*, *YHR138C*, *YKR011C*, *YNL010W*, *YSC83*

The 251 genes are manually curated into functional classes (bold) according to the summaries of their biological functions as described in the Saccharomyces Genome Database (SGD). Genes residing on the common duplicated chromosome II were not included in the analysis

The upregulated genes fall into the categories of ribosome homeostasis (66 genes), protein degradation/quality control (QC) (20 genes), protein folding/chaperones (7 genes), trafficking (18 genes), metabolism (36 genes), transcription and translation (20 genes), DNA and RNA processing (26 genes), chromosome organization/ DNA replication and repair (14 genes), cell cycle/ signaling (15), and redox homeostasis (8). The remaining 5 and 16 genes fall into other functional groups and have putative/unknown functions, respectively.

Coordinate upregulation was observed for genes encoding subunits of the 26S proteasome and of the ribosome plus their regulatory and assembly factors (Table 1, “ribosome homeostasis” and “protein degradation/QC”). For the proteasome, the known constituents of the 26S proteasome are upregulated along with associated factors and RPN4, which encodes a transcription factor that regulates proteasome genes. These were confirmed biochemically by immunoblotting for multiple strains (Additional file 10: Fig. S7). In the protein QC category, subunits of the CCT (or TRiC) chaperonin complex were upregulated across all strains disomic for chromosome II as were genes of the prefoldin complex. Similarly, we observed activation of the major chaperone and regulator genes HSP10, HCH1, PPT1, SSA2, and SSZ1 (the gene SSA3, an HSP70 chaperone involved in protein folding, is located on chromosome II and is highly upregulated in strains D1/8 and D13). The source data used for analysis in Fig. 4 is available (Additional file 11: Table S4).

In coordination with protein biogenesis and QC, protein trafficking pathways of the endomembrane system and nucleus are upregulated (Table 1). Genes encoding the signal recognition particle (SRP), involved in the first step of secretion, are upregulated in all four strains. Components of the COPI Golgi-to-ER retrograde system are also modestly activated. Components of the nuclear pore complex display increased expression (Table 1). Subunits of each RNA polymerase as well as translation initiation factors are also upregulated. While direct functional assays would be needed to determine if the upregulation of these various complexes and subunits corresponds to an enhancement of those specific protein functions, we aimed to understand if these multifaceted changes correlated to an overall combined positive effect on functional proteostasis in the aneuploid strains.

### Aneuploid strains support activities of multiple protein quality control pathways

Cells exquisitely regulate the expression of heteromeric protein complexes because excess subunits can misfold and become toxic. Aneuploidy subverts this regulation by overexpressing subunits from amplified genes, requiring QC mechanisms to recognize and degrade offending proteins. The observed regulatory changes to the protein homeostasis network and resistance to proteotoxic drugs in the aneuploid strains suggest a robust response by these aneuploid cells against proteostatic stress. To validate this view, we directly analyzed protein quality control activities at multiple levels in these strains.

**Fig. 5:**
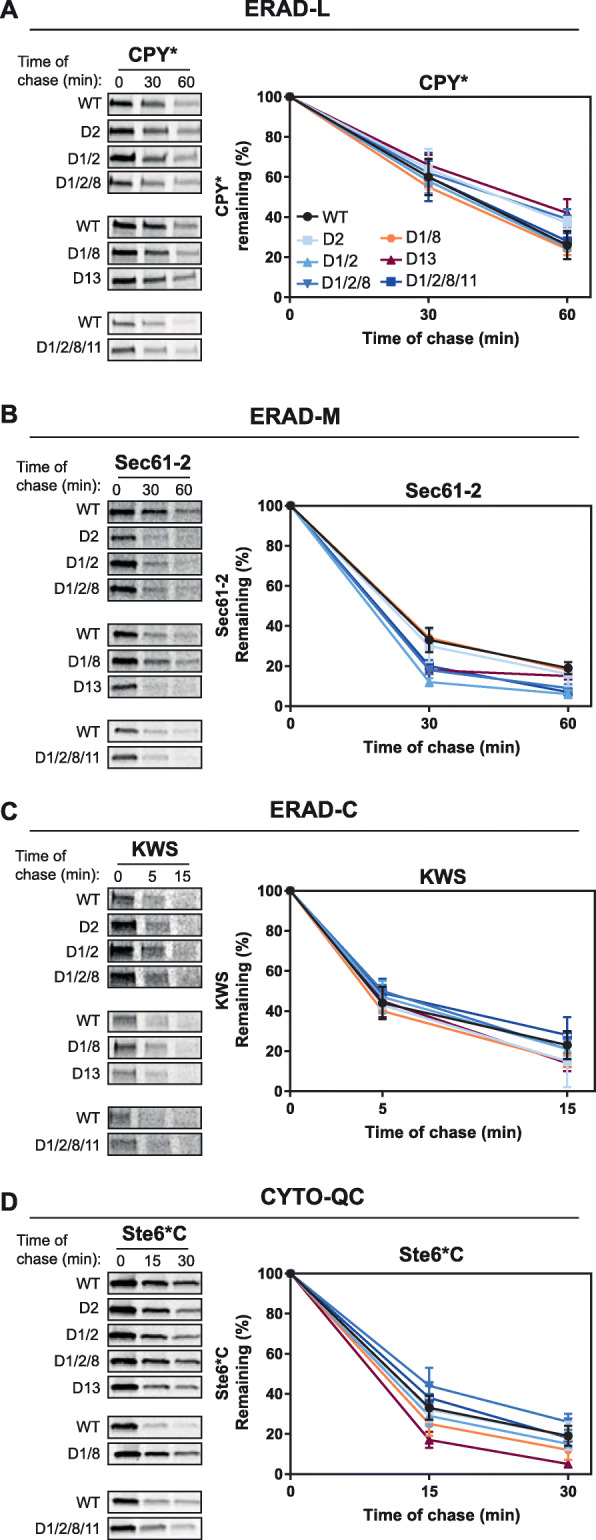
Aneuploid strains show no defects in the degradation of misfolded proteins. **a**–**d** Stability of misfolded substrate proteins in vivo subject to ER-associated degradation (ERAD) or cytosolic quality control (CytoQC) degradation pathways. Wild type (WT) and aneuploid strains were pulse-labeled for 10 min (CPY* and Sec61-2) or 5 min (KWS and Ste6*C) and chased for the times indicated. All proteins were immunoprecipitated using anti-HA antibodies and were resolved by SDS-PAGE and quantified using a phosphorimager. Representative phosphor screen scans are shown separated by experimental batches. Error bars represent the SD of three independent experiments except for WT which is displayed as an average of all experimental batches (*N* = 9 (CPY*), *N* = 10 (Sec61–2), *N* = 7 (KWS), or *N* = 9 (Ste6*C)). Turnover of **a** the ERAD luminal (ERAD-L) substrate CPY*. D1/8 (*) and D1/2/8/11 (*) are statistically different from their respective euploid controls at time 60 min (see Additional file 12: Fig. S8 for statistical comparison), **b** ERAD membrane (ERAD-M) substrate Sec61-2. D1/2 (**) and D1/2/8 (**) and D1/2/8/11 (*) are statistically different from their respective euploid controls at time 60 min, and **c** ERAD cytosolic (ERAD-C) substrate KWS. No aneuploid strains were statistically different from their respective euploid controls at time 15 min, **d** CytoQC substrate Ste6*C are shown. No aneuploid strains were statistically different from their respective euploid controls at time 30 min, Student’s *t* test: ***p < 0.001, **p < 0.01, *p < 0.05, and not significant *p* ≥ 0.05 is left unmarked. The following figure supplements are available for Fig. 5: Additional file 12: Fig. S8 and Additional file 13: Fig. S9

To compare protein QC activities of euploid and aneuploid strains, we utilized well-established model substrates of specific degradation pathways. In the secretory pathway, misfolded proteins are recognized and degraded by ER-associated degradation (ERAD). We analyzed the degradation of CPY*, a classical substrate of the ERAD-L (luminal) pathway [34, 35]. In metabolic pulse-chase experiments, CPY* is degraded more rapidly in strain D1/8, more slowly in strain D1/2/8/11, and similarly to WT in the remaining aneuploid strains (Fig. 5a, Additional file 12: Fig. S8A). We next examined the turnover of Sec61-2p, an aberrant transmembrane protein degraded by the ERAD-M (membrane) pathway [36]. Surprisingly, Sec61-2p is degraded more rapidly in D1/2, D1/2/8, and D1/2/8/11 strains and degraded as well as WT in the remaining strains (Fig. 5b, Additional file 12: Fig. S8B). We next analyzed the degradation of KWS, a type 1 membrane protein with a misfolded cytosolic domain which leads to its degradation through the ERAD-C (cytosolic) pathway [37]. Similarly, KWS is degraded at similar rates across all strains relative to (Fig. 5c, Additional file 12: Fig. S8C). For cytosolic protein QC (CytoQC), we analyzed the turnover of Ste6*C, a terminally misfolded protein that contains the misfolded cytosolic domain of Ste6-166p [38]. By metabolic pulse-chase experiments Ste6*C, a substrate of the Ubr1 CytoQC pathway exhibits no defect in degradation kinetics relative to WT (Fig. 5d, Additional file 12: Fig. S8D). Among all aneuploid strains and substrates tested (CPY*, Sec61-2, KWS, and Ste6*C), only D1/2/8/11, the strain with the highest number of excess ORFs, exhibited a modest delay in CPY* turnover (Additional file 12: Fig. S8A). When protein QC pathways are saturated, the rate of misfolded protein turnover is decreased [39–41]. Generally, all aneuploid strains degraded the ectopically, highly expressed terminally misfolded proteins either as well as, or faster, than WT indicating non-saturated QC pathways in the tolerant aneuploid strains (Additional file 12: Fig. S8A). To further validate this biochemically, we expressed a misfolded CPY^*^ variant, called D2CPY^*^, that is unable to utilize the vacuolar degradation pathway when ERAD is saturated, resulting in a stable kinetic profile when expressed under the ERAD-saturating galactose (Gal) promoter [39]. Surprisingly, of the strains tested, D2, D1/2, D1/2/8, and D1/2/8/11 strains successfully degraded Gal-D2CPY^*^ faster than the WT control, whereas it was stable in WT cells (as expected) and D1/8 (Additional file 13: Fig. S9). In this experiment, strains D2, D1/2, D1/2/8, and D1/2/8/11 all reproducibly has less intense labelling of Gal-D2CPY^*^. It is possible that lower expression of the construct in these strains allowed the degradation machinery to not become overburdened and thus degrade the construct at enhanced rates relative to WT and D1/8 which had very high, saturating levels of Gal-D2CPY^*^ expression. Regardless, the D2, D1/2, D1/2/8, and D1/2/8/11 strains did not exhibit a defect in their ability to degrade the misfolded substrate. Collectively, these data demonstrate that aneuploid strains maintain robust cytosolic and ER QC pathways even when faced with increasingly heavy substrate loads from duplicated chromosomes and ectopic expression of model misfolded substrates.

**Fig. 6:**
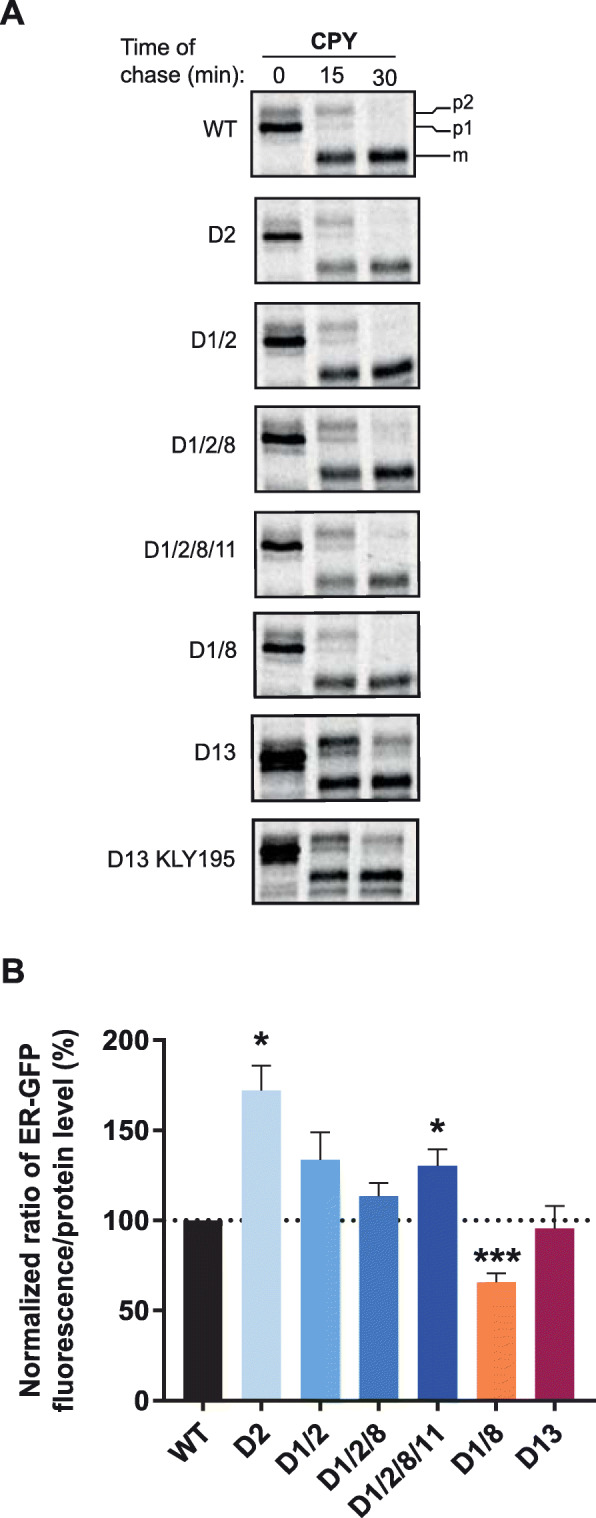
Aneuploid strains show no severe defects in protein biosynthetic capacity. **a** Biosynthesis of the endogenous protein carboxypeptidase Y (CPY) was monitored following a 5-min pulse-label. All proteins were immunoprecipitated using anti-CPY antibodies and were resolved by SDS-PAGE for wild type (WT) and aneuploid strains. The ER (p1), golgi (p2), and mature vacuolar form (m) of CPY are indicated. Three independent experiments were performed, all yielding consistent observations. Results from one representative experiment are shown. **b** Average fluorescence of ER-targeted green fluorescent protein (ER-GFP) normalized to steady-state protein levels in WT and aneuploid strains. Error bars represent the standard error of the mean (SEM) of three (D1/2/8) or four (all other strains) independent biological replicates. Welch’s *t* test: ***p < 0.001, **p < 0.01, *p < 0.05, and not significant *p* ≥ 0.05 is left unmarked. The following figure supplements are available for Fig. 6: Additional file 14: Fig. S10 and Additional file 15: Table S5

The increase of genome content directs a proportionate increase in protein synthesis, placing immediate strain on existing biosynthetic systems. Indeed, expression profiling shows a broad coordinate upregulation of components mediating translation, protein folding, trafficking, and degradation. To functionally validate these data, we studied biosynthetic capacity in these cells. First, biosynthesis of the endogenous proteins CPY and Glycophospholipid-Anchored Surface 1 (Gas1p) were monitored following a 5-min radioactive pulse-label (Fig. 6a, Additional file 14: Fig. S10). Endogenous CPY must fold properly for transport, and inter-organelle trafficking can be monitored through accumulation of the ER (p1), golgi (p2), or mature vacuolar forms of CPY (m) (Fig. 6a). Likewise, proper biosynthesis of Gas1 can be monitored through timely generation of its respective ER and plasma membrane-bound forms (Additional file 14: Fig. S10). No defects, even modest, were observed in biosynthesis (protein synthesis, folding, trafficking, post-translational modifications) of the endogenous substrates investigated in strains D2, D1/2, D1/2/8, D1/2/8/11, or D1/8 (Fig. 6a). A mild defect (relative to severe defects [25]) was observed in D13 that was not a result of the Sec24 mutations as the defect persisted in the D13 strain with WT SEC24p (KLY195).

Next, protein folding capacity in the ER was tested in WT and aneuploid strains by using an ER-targeted green fluorescent protein (ER-GFP) which is a reporter for protein folding capacity in the ER [42]. ER-GFP folds slowly in the ER and is subject to ER QC mechanisms [42]. To test the protein folding machinery further, this reporter was overexpressed using the strong constitutive *TDH3* promoter. Strains D1/2, D1/2/8, and D13 showed no deficiencies in folding based on this reporter assay (Fig. 6b). Incredibly, strain D2 had nearly twofold higher normalized fluorescence activity compared to WT, and the strain of the largest chromosomal content (D1/2/8/11) also showed modest though significant improvement in substrate folding over WT (Fig. 6b, source data provided in Additional file 15: Table S5). Conversely, strain D1/8 exhibited significantly decreased ability to fold ER-GFP indicating karyotype-specific differences. Together, these data show that the increased need for protein biosynthesis can be met functionally by broad transcriptional regulatory changes increasing protein biosynthetic capacity.

## Discussion

This study addressed the question if haploid budding yeast cells can tolerate the effects of large-scale protein imbalances, in this case brought on by aneuploidy. The isolation of stable, robust individuals from an established collection of randomly generated aneuploid strains was critical to our findings. When the original collection was generated and triploid spores were collected, there were many aneuploid progeny which were inviable or unstable (only 12.5% of viable spores were found to be isogenic, stable lines) [17]. Thus, the subset of stable, isogenic strains generated likely produced strains harboring combinations of aneuploid chromosomes that are well tolerated. We applied an unbiased approach to analyze regulatory changes at the transcriptional and translational level. These data guided direct in vivo analyses that demonstrated the functional enhancement of multiple facets of the protein homeostasis network in some strains with chromosome II duplicated, activities compromised in less tolerant cells [15, 18, 19]. These results were appealing because they address the most pressing physiological challenge in aneuploidy—mass dysregulated protein synthesis from excess chromosomes. We envision that sufficient buffering capacity of protein quality control pathways in the S288c strains with chromosome II duplicated facilitates the ability to handle the increased burden the excess chromosome places on the proteostasis network. Global expression analysis in disomic II strains demonstrated coordinate modest upregulation of protein biosynthetic and degradation pathways that may be involved, in addition to the expected pathways needed to handle excess chromosomal content and their transcription. For example, coordinate upregulation was observed for genes encoding subunits of the 26S proteasome and of the ribosome plus their regulatory and assembly factors (Table 1, “ribosome homeostasis” and “protein degradation/QC”). This is notable because these large complexes are the two core components of cellular protein homeostasis. Directly related are genes involved in protein folding and QC which were also observed to be coordinately upregulated. In conjunction with protein biogenesis and QC, protein trafficking pathways of the endomembrane system and nucleus are upregulated in disomic II strains (Table 1). This could be expected because of the increase in the synthesis of ribosomes, proteasomes, and various RNA species due to the imbalanced chromosomal content. These molecules comprise the major traffic into and out of the nucleus. Additionally, transcription and translation components such as RNA polymerase are upregulated, which is expected due to the load caused by increased chromosomal content.

These alterations are karyotype specific, as those strains without chromosome II duplicated (D1/8 and D13) rather exhibited an expression signature resembling the ESR and are sensitive to stress-inducing drugs. In contrast, those strains with chromosome II in excess do not induce stress pathways, such as the ESR or HSR, and exhibit enhanced resistance on some drugs which stress cellular homeostasis. These data suggest the sufficient cellular capacity of strains of certain karyotypes and genetic backgrounds to tolerate certain aneuploidies, and likely the alterations of the proteostasis pathways in these strains, is a consequence of the chromosome II duplication. It will be interesting and informative to understand why this chromosome in this genetic context may have this effect.

The D2, D1/2. D1/2/8, and D1/2/8/11 strains behave similarly under all conditions examined despite the stepwise increased chromosomal content and burden to the proteome. Our direct assays demonstrated WT-like or enhanced protein quality control activities without signs of proteotoxic stress, functionally supporting the broad (although mild) upregulation of key components including the proteasome, components of the ERAD, and CytoQC pathways in these strains. Previously reported aneuploidy-associated stress signatures, such as proteostasis defects, have been attributed to an *SSD1* mutation present in the common laboratory yeast strain W303 [43]. The strains used in our study from the S288C background harbor the wild type form of the SSD1 gene, and perhaps with this genetic background in combination with the karyotypes discussed here with chromosome II duplicated, these strains are inherently less sensitive to aneuploidy than other strains of different genetic backgrounds and karyotypes. Another study has also observed that some select S288c strains specifically harboring chromosome II duplication (among other chromosomes) does not activate the ESR [44].

In support of the view that the duplication of chromosome II likely plays an integral part in the enhanced capacity of the proteostasis network in these aneuploid-tolerant cells, another study conducted a genetic screen to identify tunicamycin-resistant yeast clones and found duplication of chromosome II to be associated with increased resistance to tunicamycin-associated ER-stress without induction of the UPR [45]. Additionally, in the initial studies conducted to generate 38 isogenic, stable aneuploid strains by induction of meiosis in triploids and pentaploids, there was a statistically significant enrichment for chromosome II duplication in the aneuploid progeny [17]. While it is possible this is an artifact of random meiosis I segregation, it will be interesting to explore whether this enrichment is due to this specific karyotype being more favorable toward tolerating the complex aneuploid state observed in these strains. It is possible that the collection of stable, isogenic lines is inherently enriched for strains and karyotype combinations which are more naturally more tolerant to the aneuploid state (for example beneficial chromosome duplications like chromosome II, or combinations that result in fewer toxic protein complex subunit expression imbalances). In cancer, it has also been observed that while some karyotypes and single-chromosome gains are not well tolerated, over time, cells can evolve by acquiring other additional chromosome copy number changes that provide fitness advantages [10]. This existence of both aneuploid-sensitive and aneuploid-tolerant contexts may shed light on the seeming paradox that cancer cells are both highly aneuploid yet also fiercely proliferative. Ultimately tolerance to aneuploidy-associated stresses (such as increased protein load) is likely a combination of genetic background, method of aneuploidy generation, general aneuploidy-related responses, and karyotype-specific changes. Taken together, this work underscores the importance of studying the consequences of aneuploidy and mechanisms of aneuploidy tolerance in diverse backgrounds and cell lines.

## Conclusions

We discovered that yeast strains of different karyotypes can functionally tolerate the large aneuploidy-associated burden to the proteostasis machinery. In some strains, facets of proteostasis including translation, folding, and quality control systems are enhanced without genetic changes, dosage compensation, or activation of proteotoxic stress pathways. These results indicate that proteotoxic stress is not always an obligate consequence of aneuploidy and certain karyotypes and genetic backgrounds have inherent mechanisms that mitigate the burden placed on the protein homeostasis machinery. This offers insight to the long-standing cancer conundrum whereby cancer cells are paradoxically both highly aneuploid and highly proliferative at the same time.

## Methods

### Strains and growth conditions

*Saccharomyces cerevisiae* strains used in this study originate from the S288C background (MATa ura3 his3 trp1 leu2 LYS2) and are listed in Additional file 1: Table S1 and their respective karyotypes in Additional file 2: Table S2. Plasmids and primers used in this study are provided respectively in Additional file 16: Table S6 and Additional file 17: Table S7. Mutant strains and deletion cassettes were generated using standard methods as previously described [46]. Cells were grown on rich medium (YPD), or synthetic complete (SC) media containing 2% glucose, 3% raffinose, or 2% galactose as a carbon source, yeast nitrogen base, plus the amino acids and ingredients contained in the drop out mix. Strains were cultured at 25 °C unless otherwise noted. Karyotyping of the duplicated chromosomes was performed on active cultures to ensure phenotypes observed were not due to loss of the duplicated chromosomes.

### Karyotype determination by qPCR

Karyotyping of all 16 chromosomes was carried out as described in previous studies [17]. Briefly, strains were cultured in YPD at 25 °C. Overnight cultures were normalized to an OD_600_ of 0.3, and a total of 300 μL normalized cell culture was washed twice in PBS. Cells were digested with zymolyase 20T (MP BioMedicals) for 1 h at 37 °C. qPCR-based high-throughput method was applied to measure the absolute chromosome copy numbers. 384-well qPCR plates were loaded using a Freedom Evo 150 liquid-handling robot (Tecan) and qPCR reactions were carried out in a 7900HT Fast Real-Time PCR machine (Applied Biosystems). Data were then analyzed in R using a customized script described previously [17]. The mean copy number calculated from 3 technical replicates using the probe set of the respective left chromosome arm and the mean copy number calculated from 3 technical replicates using the probe set of the respective right chromosome arm were averaged, and this mean and standard deviation (SD) from one biological replicate per strain are visualized in Fig. 1a.

### Antibodies

The following antibodies were used in this study at the indicated dilutions for western blot, or 1 μL per 708 μL for pulse-chase immunoprecipitation (IP) experiments prior to the addition of varying volumes of lysate for IP. Anti-HA mouse monoclonal antibody (HA.11) (1: 5000) was purchased from Biolegend (Cat #901515). Anti-rabbit IRDye 680 and anti-mouse IRDye 800 secondary antibodies (both 1:10000) were purchased from LI-COR Biosciences (Cat #926-68021-0.5MG, and 926-32210-0.5MG, respectively). Anti-Pgk1 mouse monoclonal antibody (1:7500) was purchased from Thermo Fisher Scientific (Cat# 459250). Anti-Kar2 rabbit polyclonal antibody (1:50000) was provided by P. Walter (University of California, San Francisco, CA). Anti-GFP mouse monoclonal antibody was purchased from Roche (Cat# 11814460001) (1:10000). Anti-Rpn5, Rpn8, Rpn9, and Rpn12 (all 1:500) were a kind gift from Daniel Finley (Harvard Medical School, Boston, MA). For pulse-chase IP, Anti-CPY rabbit polyclonal antibody was provided by Dr. Reid Gilmore (University of Massachusetts, Worcester, MA, USA) [47] and rabbit polyclonal anti-Gas1 antibody was generated by Davis Ng.

### Phenotypic characterization of aneuploid strains

Cells were grown in YPD or SC at 25 °C until they reached log phase. Equal amounts of cells were collected and reconstituted to a final OD_600_ of 0.2. Fivefold serial dilutions were spotted on synthetic complete medium with the various supplements. Plates were incubated for 3 days at 25 °C. Plates were scanned and images acquired using the same contrast and brightness settings. Cadmium chloride (99.99%), Hygromycin B from *Streptomyces hygroscopicus* (> 60%, HPLC), tunicamycin from *Streptomyces* sp., and cycloheximide were obtained from Sigma-Aldrich. The spot-tests were independently replicated three times, all with consistent observations, and representative scans from one of these experiments is shown.

### Metabolic pulse-chase assay

Metabolic pulse-chase experiments were performed as described previously [48]. Briefly, 3.0 OD_600_ units of cells were spun down and resuspended in 0.9 mL of SC media lacking methionine and cysteine. Cells were incubated at 30 °C temperature (this brief higher temperature than the normal 25 °C growth condition was used to be consistent with literature using the listed model misfolded protein substrates) for 30 min in water bath and labeled with 80 μCi of [^35^S]-methionine/cysteine for 10 min. The label was chased by adding excess of cold methionine and cysteine to a final concentration of 2 mM. Three OD units were removed at desired time points, and chase was terminated by addition of 100 μL of 100% trichloroacetic acid (TCA) to a final concentration of 10%. After protein extract preparation, proteins were immunoprecipitated with the appropriate antiserum and separated in 4–5% gradient precast gels, or 8% homemade gels (CPY and Gas1) for SDS-PAGE. Protein visualization and quantification were performed using a Typhoon 8600 scanner and ImageQuantTM TL software (GE Healthcare Biosciences). All quantified data plots reflect three independent experiments unless otherwise noted with the standard derivation (SD) indicated as error bars. A representative gel image from single experiment was shown in the figures.

### Protein extract preparation and western blot analysis

For yeast whole cell lysate, 2.0 OD_600_ units of cells were collected. Pellets were resuspended in 1 mL of 10% TCA. Cells were homogenized by the addition of 0.4 mL of 0.5-mm zirconium beads and agitated in mini-bead beater disruptor (Biospec Products) for 30 s at room temperature followed by 5-min incubation on ice. This step was repeated, and supernatant was transferred to a fresh tube. The beads were washed once with 0.4 mL 10% TCA and pooled with the saved supernatant. Supernatant was centrifuged at 14,000 rpm for 15 min at 4 °C, and the TCA precipitated pellet was resuspended in TCA suspension buffer (100 mM Tris-HCl pH 11.0, 3% SDS, 1 mM PMSF) and heated to 100 °C for 10 min. Sample was finally spun down at 14,000 rpm for 10 min at 4 °C to remove SDS insoluble debris and supernatant was collected in a new tube. Dithiothreitol (DTT) was added to 100 mM for reducing analysis. The whole cell lysate in TCA resuspension buffer was stored at − 20 °C for further experiments. The total SDS-soluble protein extract was separated on a 4–15% gradient gel by SDS-PAGE, and the resolved proteins were transferred to nitrocellulose membrane. Proteins were probed with the primary antibody followed by anti-mouse IRDye 800 and/or anti-rabbit IRDye 680 secondary antibodies. Protein of interest was visualized and quantified by Odyssey infrared imaging system (LI-COR Biosciences).

### ER-GFP-based assay for protein biosynthetic capacity

The ER-GFP-based assay was performed as described previously [42]. Briefly, the ER-GFP expression vector pWX206 [42] was introduced into WT and strains D2, D1/2, D1/2/8, D1/2/8/11, D1/8, and D13. Cells were grown to log phase, and one OD600 of cells per strain was resuspended in ice-cold PBS and filtered (30 μm). Fluorescence intensity of 30000 cells was determined by flow cytometry (CyAn ADP Analyzer, Beckman Coulter), and cells in the gated area were used for fluorescence measurement. In parallel using the same cultures, protein lysates were prepared from two OD600 units of cells and analyzed by quantitative immunoblotting to determine relative total GFP protein level using the Pgk1 or Kar2 proteins as the loading controls. As the fluorescence intensity represents folded GFP protein level, the relative folded GFP level was then calculated by normalizing fluorescence intensity with total GFP protein level determined by the quantitative immunoblotting. For Fig. 6, this ratio (normalized ratio of ER-GFP; fluorescence/protein level) determined for WT and each aneuploid strain was then normalized relative to WT (WT = 100%).

### UPR analysis and β-galactosidase reporter assay

UPRE-CYC1-LacZ reporter described by others [49] was transfected into aneuploid strains and the WT control. Cells were cultured at 25 °C until they reached optical density of 0.2 OD_600_/mL. Next, cultures were treated with tunicamycin (TM) at the concentration 1 μg/mL for 1 h prior to harvesting cells, or mock-incubated. Cells were analyzed as described previously [25]. Briefly, two OD_600_ units were collected and washed with Z buffer (60 mM Na_2_HPO_4_, 40 mM NaH_2_PO_4_, 10 mM KCl, 1 mM MgSO_4_, 50 mM β-mercaptoethanol). Cells were resuspended in 50 μL of Z buffer, 20 μL of CHCl_3_, and 20 μL of 0.1% SDS. The reaction mixture was incubated with 2 mg/mL o-nitrophenyl-β-galactopyranoside (Sigma) at 30 °C for a maximum of 10 min. The reaction was quenched with Na_2_CO_3_, and the reaction period was noted. The absorbance at *λ* = 420 nm of the supernatant was measured, and β-galactosidase activity was calculated in Miller units where Miller units = 1000 × (OD420 – 1.75 × OD550)/(2*t* × OD600) and *t* = time (min). The β-galactosidase activities of aneuploids were normalized to the activity of the WT control.

### *HAC1* splicing

Exponentially growing cells were treated for 1 h with tunicamycin (TM) at the concentration 1 μg/mL. Control samples and TM-treated samples were harvested, and the total RNA was extracted using the RNeasy Mini kit with On-Column DNase digestion (Qiagen). Superscript III kit (Invitrogen) was used for the first-strand cDNA synthesis from 2 μg of total RNA per sample. *HAC1u* (unspliced) and *HAC1s* (spliced) cDNAs were amplified by PCR, the PCR products were then run on 2% agarose gels, and the SYBR-stained images were quantified. *ACT1* mRNA was used as an internal control for the normalization.

### Ribosome profiling

For the preparation of ribosome-protected fragments and total RNA libraries, Deep Sequencing Truseq™ Ribosome Profiling kit (Illumina) was used for obtaining ribosome-protected RNA fragments using size-exclusion chromatography columns. Both RPF and total mRNA input fragments were cloned according to the manufacturer protocol. Sequencing libraries were sequenced using an Illumina HiSeq 2000 sequencer. One biological replicate each of strains D2, D1/2, D1/2/8, and D13 (KLY196) were prepared for RNA-seq and ribosome profiling analysis, and two biological replicates each of D1/2/8/11, D1/8, and D13 (KLY195) were prepared. Strains D2 and D1/2 were prepared together along with a WT strain and log_2_ ratios for D2 and D1/2 were compared to those of this biological replicate of the haploid euploid strain (WT). The remaining strains were analyzed along with another WT strain prepared together and log_2_ ratios for those strains were compared to those of this WT biological replicate. For gene expression plots (Fig. 2 and Additional file 5: Fig. S3), one randomly chosen representative biological replicate of each strain was used for analysis and the data from all strains and replicates has been deposited in NCBI’s Gene Expression Onmibus (GEO) under series number GSE140733. No outliers were removed from Fig. 2 and Additional file 5: Fig. S3; however, the *y* axis of all graphs was limited to between − 1 and + 2 log_2_ fold change, and thus, datapoints outside of this range will be outside of the plot area. All strains were grown in YPD at 25 °C.

For RNA-seq and ribosome profiling analysis, sequencing adapters were trimmed using Cutadapt [50], then mapped to *S. cerevisiae* SacCer3 full-length mRNAs using Bowtie [51] allowing for no mismatches and only counting reads that fell within the annotated coding sequence toward ribosome footprinting-related metrics (for number of mapped reads see Additional file 18: Table S8). Abundance of each mRNA and its translation was then quantified as reads per kilobase of coding sequences per million mapped reads in the library (RPKM). To enable straightforward assessment of the impact of chromosome duplications, all RPKM values were subsequently normalized such that the median mRNA in a nonduplicated chromosome in an aneuploid strain was equal to the RPKM of that mRNA in a euploid strain. This was accomplished by calculating the median of RPKM (aneuploid)/RPKM (euploid) for all genes on nonduplicated chromosomes, then dividing all RPKM values in the aneuploid strain by this ratio. Genes residing on mitochondrial DNA (chromosome Mito) were not included in subsequent analysis. Representative correlations of mRNA log2 foldchange RPKM values of several aneuploid strains relative to WT compared to one another strain relative to WT are available in Additional file 19: Fig. S11.

### Whole genome re-sequencing

Whole genome re-sequencing was performed on the isogenic wild type strain, as well as the aneuploid strains D2, D1/2, D1/2/8, and D13 (KLY196). Cultures were grown overnight in YPD at 25 °C and were harvested when reaching mid-logarithmic phase. Genomic DNA was isolated using the phenol-chloroform method as previously described [52]. DNA libraries were prepared according to manufacturer specifications (Ion Xpress™ Plus Fragment Library Kit), and DNA Libraries were sequenced using Ion Torrent sequencing on an Ion Proton System (ThermoFisher Scientific). Reads (approximately 150 bp) were aligned to the *Saccharomyces cerevisiae* S288C reference genome version 64 (UCSC sacCer3) using Novocraft novoalign (version 3.4). Alignments at duplicate positions were discarded using Picard MarkDuplicates (version 1.127). The WT sample had over 26 M reads and 4 Gb of sequence data, while the mutant samples had 10–15 M reads and 1.5–1.9 Gb. In all cases, the fraction of base qualities over 20 was 86–88%, with median quality about 26. Mean sequence depth for WT was 155X, while the mutants ranged from 71X to 80X (Additional file 20: Table S9).

To examine the copy number ratios across each chromosome, normalized read counts (RPKM values) were collected for 1-kb intervals across the genome using the BioToolBox program get_datasets (version 1.36) for each strain. Log_2_ ratios were generated between the values from the aneuploid strains and WT, and graphs generated with GraphPad Prism (version 6).

### Identification of SNPs

Variants were called with Samtools (version 1.3) [53] and VarScan (version 2.4.0 [54], minimum base quality 15, minimum mapping quality 15). Variants were further filtered for false positives with the VarScan fpfilter (max-var-mmqs 350, max-mmqs-diff 250, min-basequal 15, min-mapqual 15) using alignment statistics collected with bam-readcount (version 0.7.4). Homozygous haploid variants were further filtered for a minimum variant frequency of 70% to reduce noise. However, we also considered the possibility of heterozygous aneuploid variants on the duplicated chromosomes, using a 40% cutoff.

To identify variants unique to the aneuploidy strains, the variants were intersected with those from the wild type strain using bedtools (version 2.25.0) requiring perfect reciprocal match [55]. Variants were annotated using the Ensembl Variant Effect Predictor tool (release 84) [56]. Mutant genes were identified by containing a variant that generates a codon change, occurs at a high frequency, and is unreported in the Ensembl annotation.

No newly acquired (not present in euploid parental strain), non-clonal mutations were found to have arisen in coding regions during propagation in strains D2 or D1/2. Strains D1/2/8 and D13 have the previously published missense mutation in *CAK1* (L147F) that was inherited from the parental strain U3 (RLY4708) rather than acquired through propagation [17]. In addition to the *CAK1* mutation, strain D13 (KLY196) has a missense mutation (M266R) in *SEC24*. Both of these mutations were reversed to the wild type sequences in the D13 strain KLY195 (Additional file 1: Table S1). Additionally, strain D1/2/8 acquired a C➔A mutation in a non-coding region of chromosome XIII (position 917993) upstream of YMR321C. In strain D2, there is an A➔T mutation at position 393858 on chromosome X, and in strain D1/2, a T➔A mutation at position 212540 on chromosome IV which are both intergenic regions.

### Statistical analysis

Statistic evaluation was performed in Microsoft Excel or GraphPad prism software. Specific statistical tests used are provided in figure legends. For pulse-chase experiments (Fig. 5 and Additional file 12: Fig. S8 and Additional file 13: Fig. S9), two-tailed, unpaired Student’s *t* test was performed to evaluate the statistical significance of differences between two strains (aneuploid versus euploid control, *n* = 3 of each) at the final pulse-chase timepoint using Graphpad Prism software. Significance level is defined as *p* ≥ 0.05 not significant (n.s. or blank), *p < 0.05, **p < 0.01, ***p < 0.001. For the average fluorescence of ER-targeted green fluorescent protein (ER-GFP) normalized to steady-state protein levels in WT and aneuploid strains (Fig. 6b), two-tailed, Welch’s *t* test was performed (differences in sample size between some groups) to evaluate the statistical difference between two means (aneuploid versus euploid control). For this test, significance level is defined as *p* ≥ 0.05 not significant (blank), *p < 0.05, **p < 0.01, ***p < 0.001.

## Supplementary information

**Additional file 1: Table S1.** *Saccharomyces cerevisiae* strains used in this study.

**Additional file 2: Table S2.** Karyotypes of euploid and aneuploid *Saccharomyces cerevisiae* strains.

**Additional file 3: Figure S1.** Whole genome sequencing analysis of copy number variation of the aneuploid strains D2, D1/2, D1/2/8 and D13. From whole genome sequencing analysis log2 ratios between the aneuploid strains and WT RPKM values were calculated in 1 Kb windows across the genome and represented as dot plots across each of the 16 chromosomes to visualize the copy number.

**Additional file 4: Figure S2.** Enhanced resistance of various aneuploids to stress-inducing drugs is not due to duplication of PDR3 or PCA1 genes or a complete defect in drug import. **(A-B)** Proliferation of WT, aneuploid or aneuploid knock-down strains determined by spot test under the same conditions as in Fig. 1D in the presence of tunicamycin (TM, 1 μg/mL), hygromycin B (HygB, 0.5 mg/mL), or cadmium chloride (CdCl_2_, 50 μM). **(A)** One duplicated copy of the *PDR3* gene (located on duplicated chromosome 2) was replaced by the KanMX cassette in D2 and D1/2 (D2 *Δpdr3* and D1/2 *Δpdr3* respectively). The number of copies of the *PDR3* gene present in these strains is thus returned to one and is no longer duplicated. **(B)** One duplicated copy of the *PCA1* gene (located on chromosome 2) was replaced by the KanMX cassette in D2 (D2 Δpca1) as described above. Two independent experiments were performed and representative scans are shown. **(C)** Effects of tunicamycin on the glycosylation of CPY in wild type (WT) and aneuploid strains. Cells were grown overnight in liquid culture to mid-log phase then were collected and resuspended in synthetic complete (SC) media containing a final concentration 1 μg/mL tunicamycin (TM) (+) or an equal volume of vehicle control DMSO (−) and incubated at 25 °C with rotation for 8 h. Three independent experiments were performed and a representative image from one experiment is shown. ER (p1), golgi (p2) and CPY lacking 1, 2, 3, or 4 glycans (underglycosylated forms) are indicated.

**Additional file 5: Figure S3.** Genes encoded on duplicated chromosomes undergo efficient transcription in tolerant aneuploid yeast strains. Relative levels of mRNA (left) ribosomal footprint (FP) (middle) or ribosome density (RD) (right) in aneuploid strains compared to the euploid control cells were analyzed by RNA-Seq and ribosome profiling. Each gene is shown as a circle plotted at its log2 fold change (FC) in the aneuploid strains relative to the euploid control with duplicated chromosomes highlighted in red.

**Additional file 6: Figure S4.** Aneuploid strains can handle aneuploidy-associated ER protein burden without acute, broad activation of the UPR. **(A)** Genes involved in the unfolded protein response (UPR) (gene list from [25]) were identified from mRNA-seq (mRNA) and ribosome profiling (FP) datasets for wild type (WT), and aneuploid strains. The columns labeled with _1 and _2 indicate respective biological replicates of a given strain and only nonduplicated genes were included in the analysis. The average log2 fold change (FC) expression levels in the aneuploid strains relative to the WT of genes listed were calculated (bargraph). **(B)** Mean UPR activity in WT and D2, D1/2 and D1/2/8 strains from three independent experiments (bars indicate SEM) measured by a β-galactosidase reporter assay following treatment with 2.5 μg/mL tunicamycin (TM) or vehicle control. **(C)** HAC1 splicing in WT and aneuploid strains D2, D1/2 and D1/2/8 was analyzed by measuring normal levels of spliced HAC1 compared to levels following treatment with TM, an inducer of acute UPR. Cells were treated with 1 μg/ml TM (+TM) or mock-incubated with vehicle control DMSO (−TM), then total RNA was isolated and subjected to RT-PCR to amplify the spliced (S) and unspliced (U) HAC1 products. Fragments were fractionated by agarose-gel electrophoresis and visualized by SYBR staining (left). Plot from one independent experiment shows the quantification of the spliced HAC1 relative to the total HAC1 level (right). **(D)** Indicated strains with the deletion of IRE1 were spotted at 5-fold serial dilution on SC media or SC supplemented with TM at indicated concentrations. Images from one biological replicate were recorded after three days at 25 °C.

**Additional file 7: Figure S5.** Aneuploid strains share a response to oxidative stress. **(A)** Genes involved in oxidative stress response (OSR, gene list from [31]) were identified from mRNA-seq (mRNA) and ribosome profiling footprint (FP) datasets. Only genes residing on nonduplicated chromosomes and for which complete datasets were available for all strains were included in the heatmap. The columns labeled with _1 and _2 indicate respective biological replicates of a given strain. The average log_2_ fold change (FC) mRNA and FP expression levels in the aneuploid strains relative to the WT of all nonduplicated genes involved in a given response were calculated (bar graphs). **(B)** Genes involved in YAP1/SKN7-dependent oxidative stress response (gene list from [32]) were identified from mRNA and FP datasets. **(C-D)** Genes involved in the hydrogen peroxide (H_2_O_2_)-treatment oxidative stress response pathway (gene list from [33]). Genes were separated in two groups: those targets typically induced during H_2_O_2_-dependent oxidative stress response **(C)** and those repressed **(D)**.

**Additional file 8: Table S3.** RNA-seq and ribosome footprints of ESR and HSR-related genes in aneuploid strains.

**Additional file 9: Figure S6.** GO Term enrichment of genes downregulated at the ribosomal footprint level. GO-term enrichment analysis with bonferroni correction applied to genes residing on nonduplicated or duplicated chromosomes that had at least 1.5 fold (0.585 log_2_ fold change) decrease in ribosome-protected footprint (FP) levels relative to euploid control for each strain. The columns labeled with _1 and _2 indicate respective biological replicates of a given strain. *P*-values were calculated from hypergeometric tests following enrichment of GO terms relating to GO Biological Process Classification obtained at http://funspec.med.utoronto.ca/.

**Additional file 10: Figure S7.** Aneuploid strains have elevated steady-state proteasomal subunit protein levels. **(A)** Steady-state levels of Rpn5, Rpn8, Rpn9, and Rpn12 proteins were measured by Western blot analysis in wild type (WT) and aneuploidy strains D2, D1/2 and D1/2/8. Proteins were resolved by SDS-PAGE and probed by immunoblotting using anti-Rpn5, anti-Rpn8, anti-Rpn9, or anti-Rpn12 antibodies. Endogenous Pgk1 or Kar2 were detected from the same membranes as a loading control using anti-Pgk1 or anti-Kar2 antibodies. **(B)** Quantification of indicated RPN protein levels based on the western blot shown in **(A)** with the relative protein levels normalized to the indicated loading control (one biological replicate performed for each strain and each respective probe).

**Additional file 11: Table S4.** List of genes analyzed for upregulated GO-term enrichment analysis.

**Additional file 12: Figure S8.** No defect in misfolded protein degradation capacity in aneuploid cells. **A-D)** Stability of misfolded substrate proteins in vivo subject to ER-associated degradation (ERAD) or cytosolic quality control (Cyto-QC) degradation pathways. Graphs corresponding to each batch (3 independent experiments per batch) are shown. Wild type (WT) and aneuploid strains were pulse-labeled for 10 min (CPY* and Sec61–2) or 5 min (KWS and Ste6*C) and chased for the times indicated. All proteins were immunoprecipitated using anti-HA antibodies and were resolved by SDS-PAGE and quantified using a phosphorimager. Representative phosphor screen scans are shown. Error bars represent the SD of three independent experiments. Turnover of **(A)** the ERAD-Luminal (ERAD-L) substrate CPY*, **(B)** ERAD-Membrane (ERAD-M) substrate Sec61–2, **(C)** ERAD-Cytosolic (ERAD-C) substrate KWS, **(D)** Cyto-QC substrate Ste6*C are shown. Student’s t test: *** *p* < 0.001, ** *p* < 0.01, * *p* < 0.05, and not significant *p* ≥ 0.05 is left unmarked.

**Additional file 13: Figure S9.** Enhanced degradation capacity in some aneuploid. Turnover of the degradation-deficient GAL-D2CPY* was monitored following pulse-labeling for 10 min as described previously in WT and aneuploid strains D2, D1/2, D1/2/8, D1/2/8/11 and D1/8 (data represent 3 independent experiments). Strains were grown overnight in Raffinose-containing media then induced for 4 h in galactose-containing media prior to chase for GAL-D2CPY* analysis. Student’s t test: *** p < 0.001, ** p < 0.01, * p < 0.05, and not significant p ≥ 0.05 is left unmarked.

**Additional file 14: Figure S10.** Aneuploid strains do not exhibit protein folding capacity defect or trafficking delay in aneuploid cells. Biosynthesis of the endogenous protein Gas1 was monitored following a 5 min pulse-label. Chase samples were taken at the time points indicated and Gas1 protein was immunoprecipitated using anti-Gas1 antibodies, followed by separation by SDS-PAGE and visualization and quantification by phosphorimager analysis. The ER (ER Gas1) and golgi/plasma membrane (Golgi/PM Gas1) forms are indicated in wild type (WT) and aneuploid strains D2, D1/2 and D1/2/8. Three independent experiments were performed, all yielding similar observations. Results from one representative experiment is shown.

**Additional file 15: Table S5**. Normalized ratio of ER-GFP fluorescence/protein level.

**Additional file 16: Table S6.** Plasmids used in this study.

**Additional file 17; Table S7.** Primers used in this study for RT-qPCR assays.

**Additional file 18: Table S8.** Mapped reads from RNA-Seq and ribosome profiling.

**Additional file 19: Figure S11.** The mRNA log2 fold change of one strain relative to WT compared to another strain relative to WT. mRNA log2 fold changes in one strain compared to another strain, with each genes colored according to the chromosome in which it is located. Red dots are on a chromosome duplicated only in the x axis strain, blue dots are on a chromosome duplicated only on the y axis strain, purple dots are duplicated in both.

**Additional file 20: Table S9.** Whole genome requencing read depth.

## Data Availability

The RNA sequencing and ribosome profiling datasets supporting the conclusions of this article are available in the NCBI’s Gene Expression Onmibus (GEO), under series number GSE140733 (https://www.ncbi.nlm.nih.gov/geo/query/acc.cgi?acc=GSE140733) [57]. Whole genome re-sequencing datasets supporting the conclusions of this article are available in NCBI’s Sequence Read Archive (SRA) database under BioProject accession number PRJNA590310 (https://www.ncbi.nlm.nih.gov/Traces/study/?acc=PRJNA590310%20) [58]. The datasets supporting the conclusions of this article are included within the article and its additional file(s).

## Abbreviations

CdCl_2_: Cadmium chloride
CHX: Cycloheximide
CPY: Carboxypeptidase Y
CytoQC: Cytosolic protein quality control
“D”: Disome
ER: Endoplasmic reticulum
ERAD: ER-associated degradation
ERAD-C: ERAD cytosolic
ERAD-L: ERAD luminal
ERAD-M: ERAD membrane
ESR: Environmental stress response
FC: Fold change
FP: Footprint (ribosome-protected footprint)
GFP: Green fluorescent protein
GO term: Gene ontology term
HSR: Heat shock response
HygB: Hygromycin B
OD: Optical density
OSR: Oxidative stress response
QC: Quality control
RNA-Seq: RNA sequencing
RPKM: Reads per kilobase of transcript, per million mapped reads
SC: Synthetic complete
SD: Standard deviation
SEM: Standard error of the mean
SRP: Signal recognition particle
TCA: Trichloroacetic acid
TM: Tunicamycin
UPR: Unfolded protein response
WT: Wild type
YPD: Yeast extract peptone dextrose
