# Supplementary figures and images for: Aneuploidy-induced proteotoxic stress can be effectively tolerated without dosage compensation, genetic mutations, or stress responses
Source: BMC Biol. 2020 Sep 8;18:117. doi: 10.1186/s12915-020-00852-x (PMC7487686; doi:10.1186/s12915-020-00852-x)

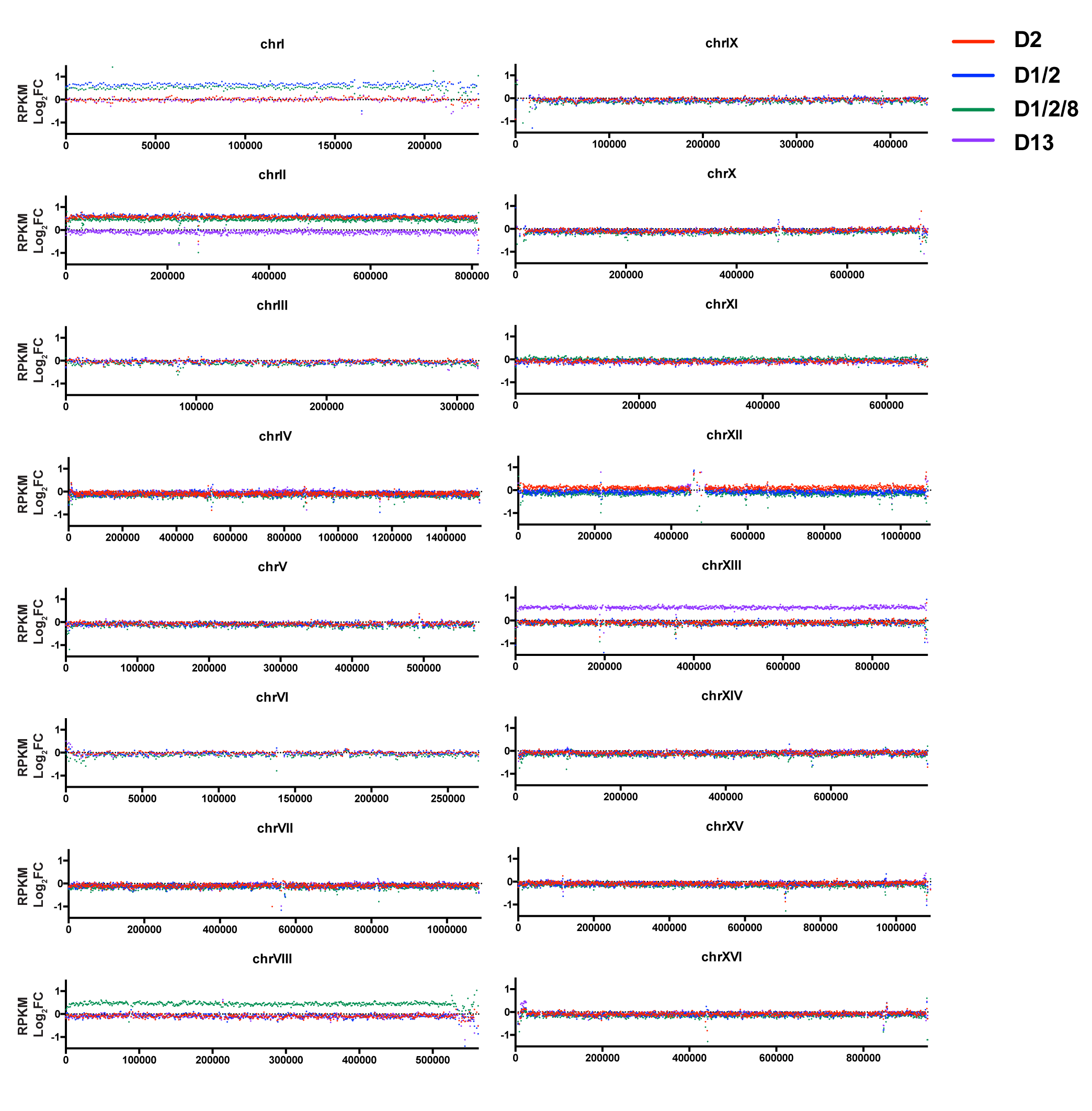

Supplement: Supplementary file 3 — Additional file 3: Figure S1. Whole genome sequencing analysis of copy number variation of the aneuploid strains D2, D1/2, D1/2/8 and D13. From whole genome sequencing analysis log2 ratios between the aneuploid strains and WT RPKM values were calculated in 1 Kb windows across the genome and represented as dot plots across each of the 16 chromosomes to visualize the copy number. [file 12915_2020_852_MOESM3_ESM.tif]

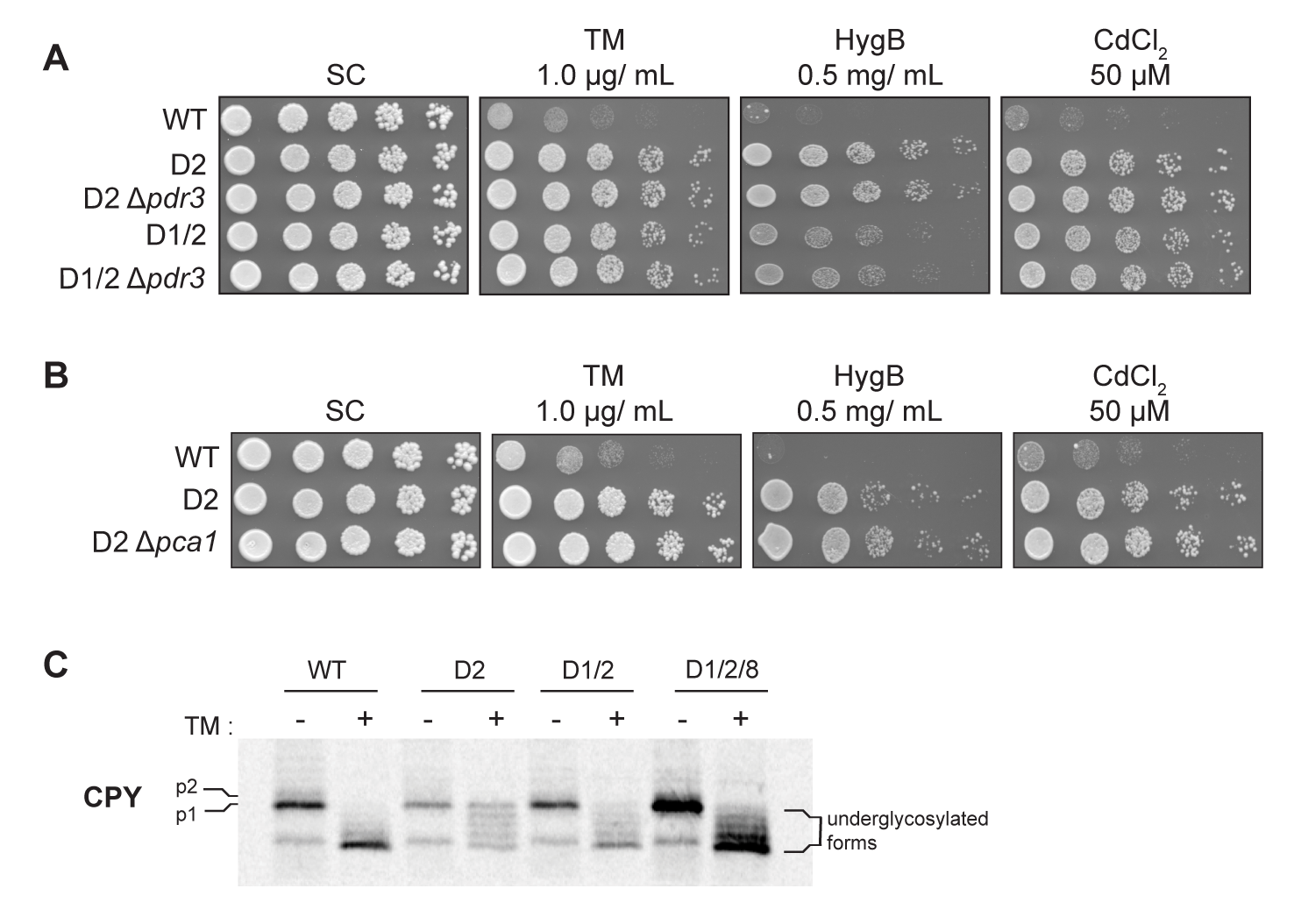

Supplement: Supplementary file 4 — Additional file 4: Figure S2. Enhanced resistance of various aneuploids to stress-inducing drugs is not due to duplication of PDR3 or PCA1 genes or a complete defect in drug import. (A-B) Proliferation of WT, aneuploid or aneuploid knock-down strains determined by spot test under the same conditions as in Fig. 1D in the presence of tunicamycin (TM, 1 μg/mL), hygromycin B (HygB, 0.5 mg/mL), or cadmium chloride (CdCl2, 50 μM). (A) One duplicated copy of the PDR3 gene (located on duplicated chromosome 2) was replaced by the KanMX cassette in D2 and D1/2 (D2 Δpdr3 and D1/2 Δpdr3 respectively). The number of copies of the PDR3 gene present in these strains is thus returned to one and is no longer duplicated. (B) One duplicated copy of the PCA1 gene (located on chromosome 2) was replaced by the KanMX cassette in D2 (D2 Δpca1) as described above. Two independent experiments were performed and representative scans are shown. (C) Effects of tunicamycin on the glycosylation of CPY in wild type (WT) and aneuploid strains. Cells were grown overnight in liquid culture to mid-log phase then were collected and resuspended in synthetic complete (SC) media containing a final concentration 1 μg/mL tunicamycin (TM) (+) or an equal volume of vehicle control DMSO (−) and incubated at 25 °C with rotation for 8 h. Three independent experiments were performed and a representative image from one experiment is shown. ER (p1), golgi (p2) and CPY lacking 1, 2, 3, or 4 glycans (underglycosylated forms) are indicated. [file 12915_2020_852_MOESM4_ESM.tif]

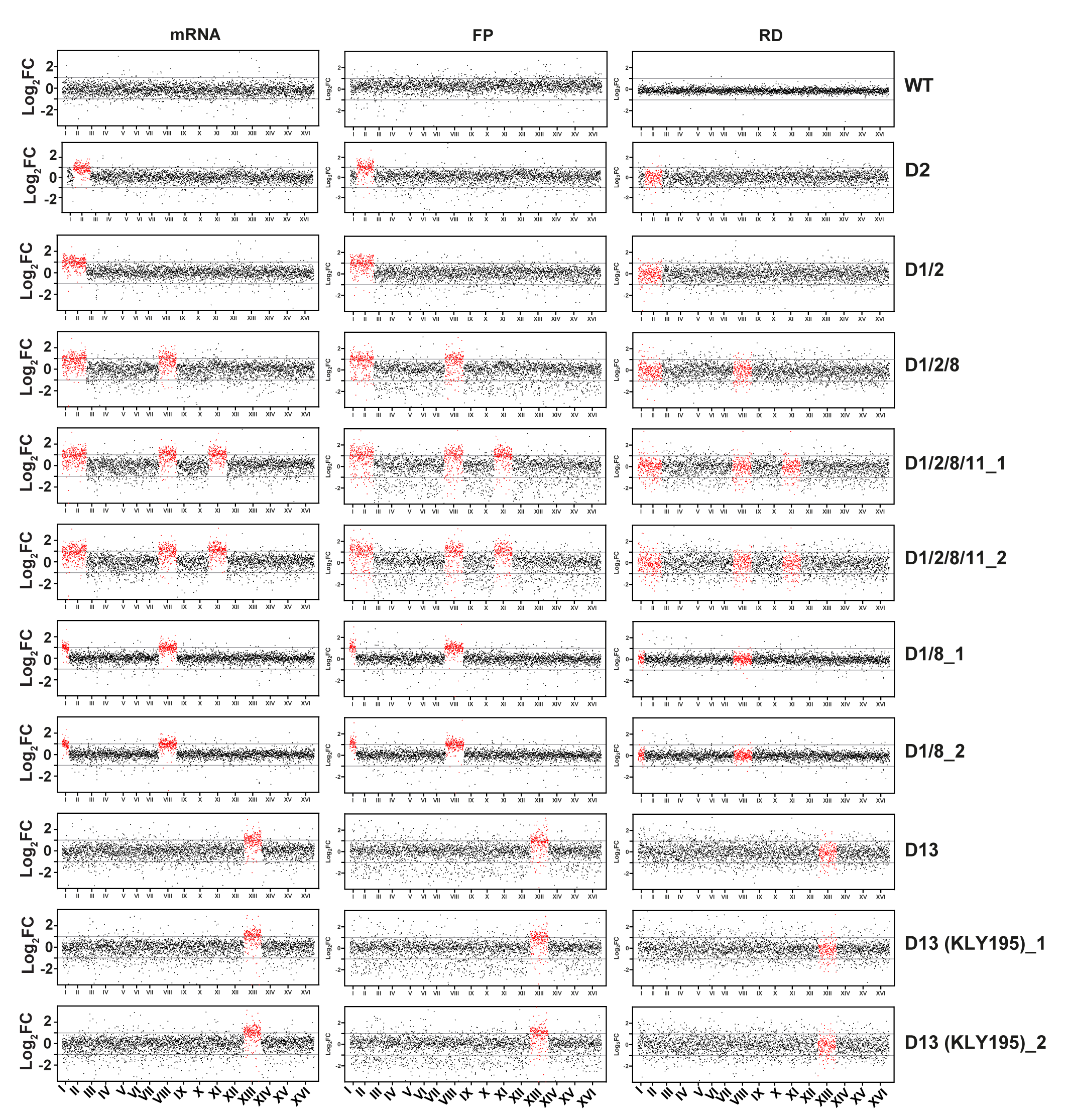

Supplement: Supplementary file 5 — Additional file 5: Figure S3. Genes encoded on duplicated chromosomes undergo efficient transcription in tolerant aneuploid yeast strains. Relative levels of mRNA (left) ribosomal footprint (FP) (middle) or ribosome density (RD) (right) in aneuploid strains compared to the euploid control cells were analyzed by RNA-Seq and ribosome profiling. Each gene is shown as a circle plotted at its log2 fold change (FC) in the aneuploid strains relative to the euploid control with duplicated chromosomes highlighted in red. [file 12915_2020_852_MOESM5_ESM.tif]

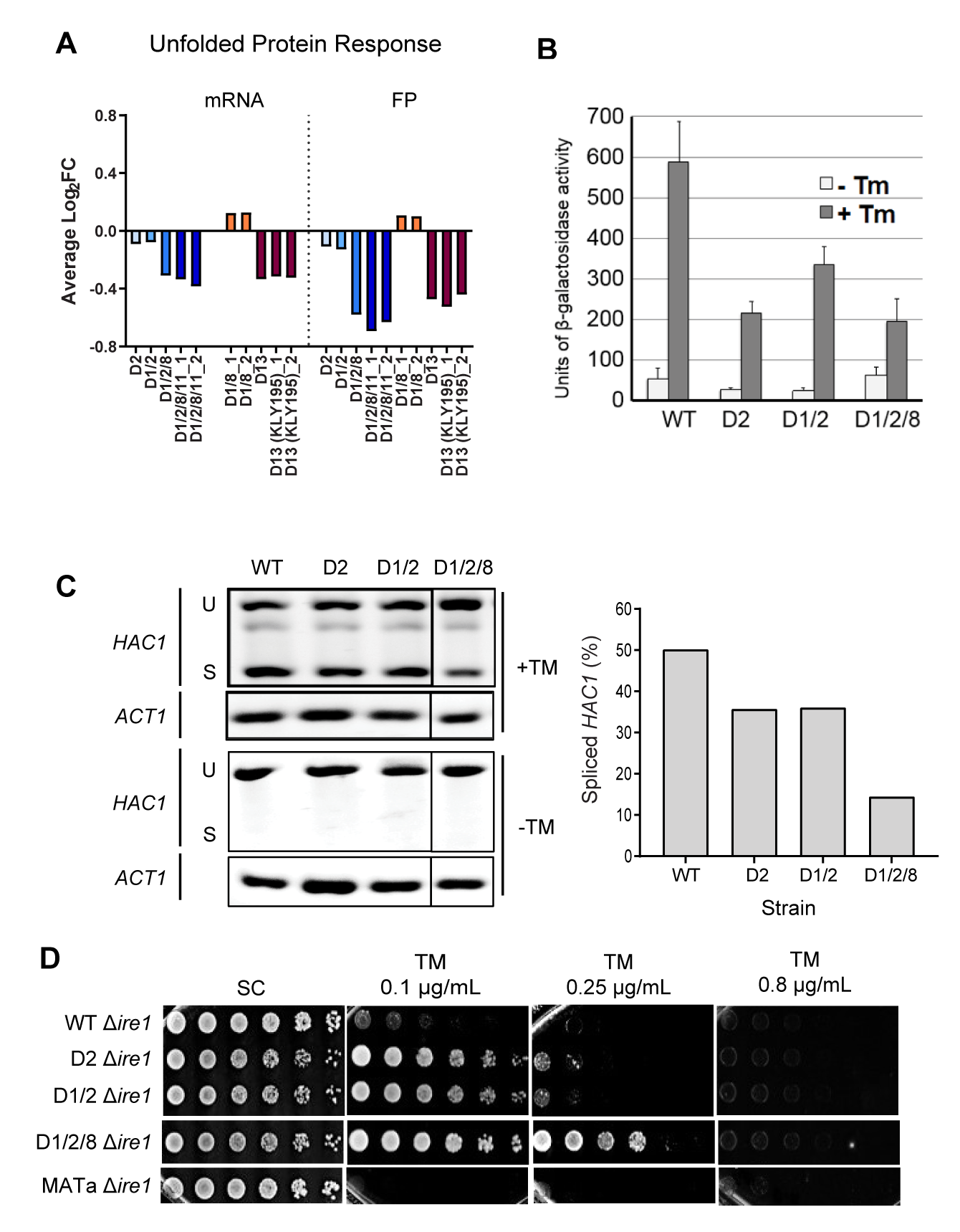

Supplement: Supplementary file 6 — Additional file 6: Figure S4. Aneuploid strains can handle aneuploidy-associated ER protein burden without acute, broad activation of the UPR. (A) Genes involved in the unfolded protein response (UPR) (gene list from [25]) were identified from mRNA-seq (mRNA) and ribosome profiling (FP) datasets for wild type (WT), and aneuploid strains. The columns labeled with _1 and _2 indicate respective biological replicates of a given strain and only nonduplicated genes were included in the analysis. The average log2 fold change (FC) expression levels in the aneuploid strains relative to the WT of genes listed were calculated (bargraph). (B) Mean UPR activity in WT and D2, D1/2 and D1/2/8 strains from three independent experiments (bars indicate SEM) measured by a β-galactosidase reporter assay following treatment with 2.5 μg/mL tunicamycin (TM) or vehicle control. (C) HAC1 splicing in WT and aneuploid strains D2, D1/2 and D1/2/8 was analyzed by measuring normal levels of spliced HAC1 compared to levels following treatment with TM, an inducer of acute UPR. Cells were treated with 1 μg/ml TM (+TM) or mock-incubated with vehicle control DMSO (−TM), then total RNA was isolated and subjected to RT-PCR to amplify the spliced (S) and unspliced (U) HAC1 products. Fragments were fractionated by agarose-gel electrophoresis and visualized by SYBR staining (left). Plot from one independent experiment shows the quantification of the spliced HAC1 relative to the total HAC1 level (right). (D) Indicated strains with the deletion of IRE1 were spotted at 5-fold serial dilution on SC media or SC supplemented with TM at indicated concentrations. Images from one biological replicate were recorded after three days at 25 °C. [file 12915_2020_852_MOESM6_ESM.tif]

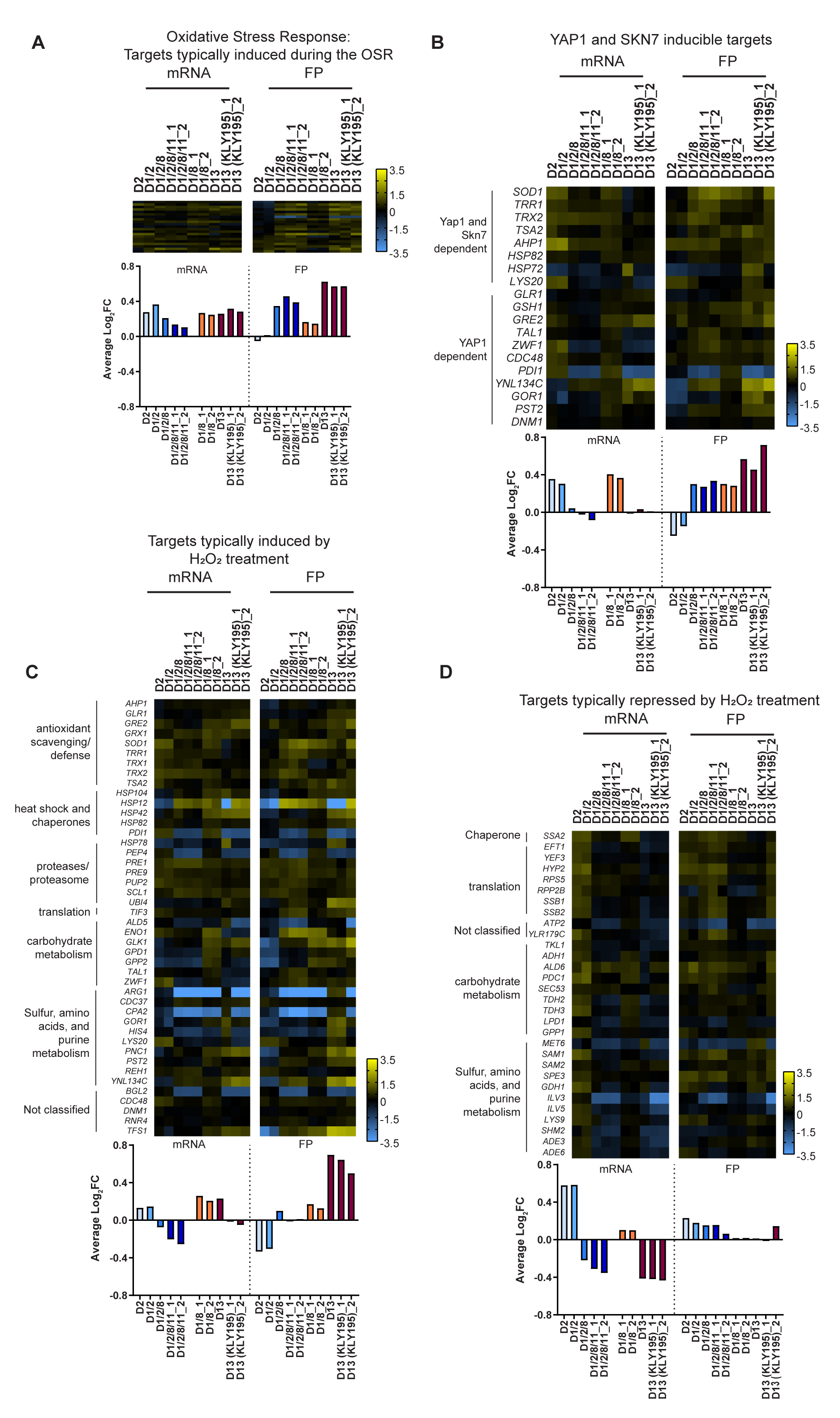

Supplement: Supplementary file 7 — Additional file 7: Figure S5. Aneuploid strains share a response to oxidative stress. (A) Genes involved in oxidative stress response (OSR, gene list from [31]) were identified from mRNA-seq (mRNA) and ribosome profiling footprint (FP) datasets. Only genes residing on nonduplicated chromosomes and for which complete datasets were available for all strains were included in the heatmap. The columns labeled with _1 and _2 indicate respective biological replicates of a given strain. The average log2 fold change (FC) mRNA and FP expression levels in the aneuploid strains relative to the WT of all nonduplicated genes involved in a given response were calculated (bar graphs). (B) Genes involved in YAP1/SKN7-dependent oxidative stress response (gene list from [32]) were identified from mRNA and FP datasets. (C-D) Genes involved in the hydrogen peroxide (H2O2)-treatment oxidative stress response pathway (gene list from [33]). Genes were separated in two groups: those targets typically induced during H2O2-dependent oxidative stress response (C) and those repressed (D). [file 12915_2020_852_MOESM7_ESM.tif]

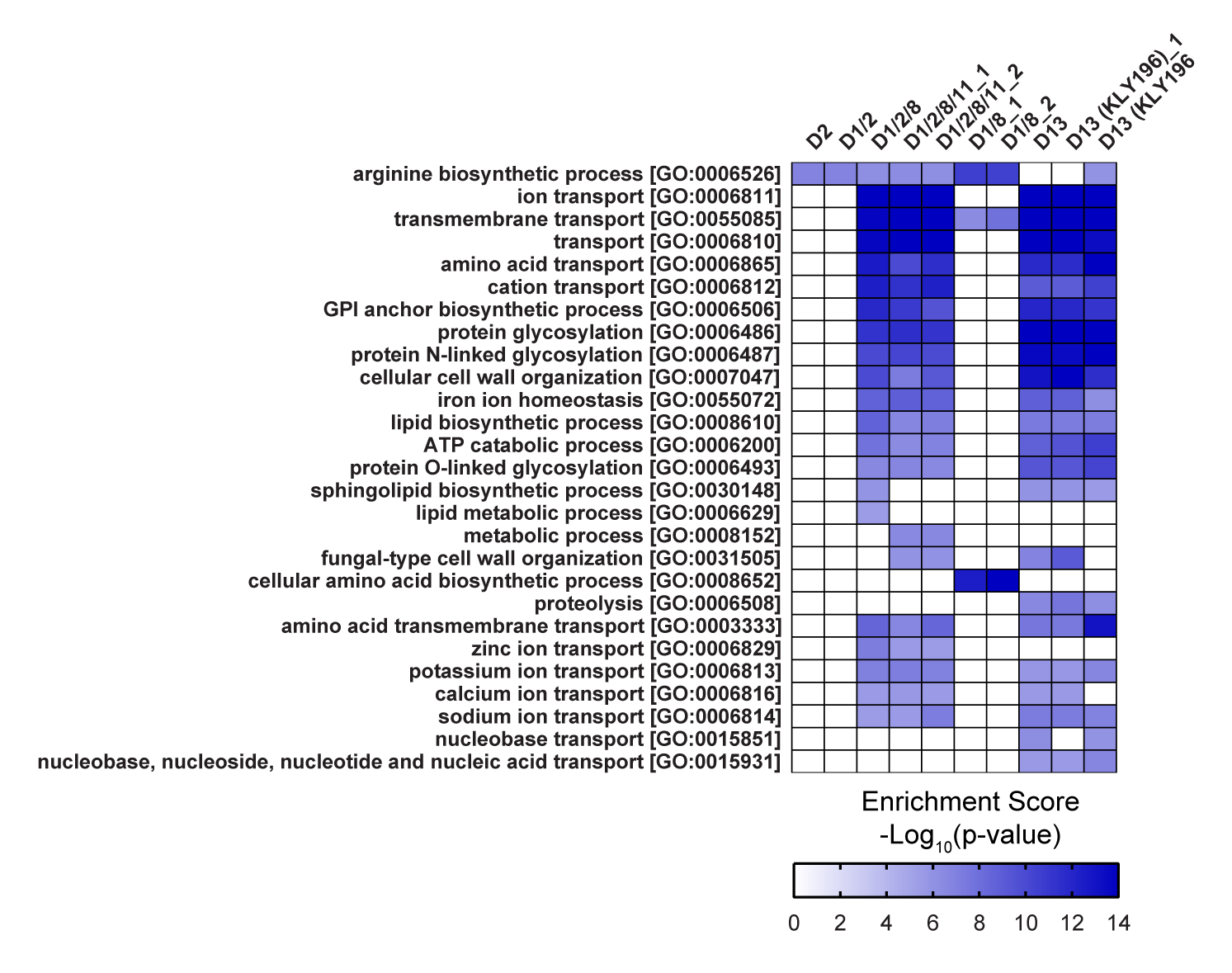

Supplement: Supplementary file 9 — Additional file 9: Figure S6. GO Term enrichment of genes downregulated at the ribosomal footprint level. GO-term enrichment analysis with bonferroni correction applied to genes residing on nonduplicated or duplicated chromosomes that had at least 1.5 fold (0.585 log2 fold change) decrease in ribosome-protected footprint (FP) levels relative to euploid control for each strain. The columns labeled with _1 and _2 indicate respective biological replicates of a given strain. P-values were calculated from hypergeometric tests following enrichment of GO terms relating to GO Biological Process Classification obtained at http://funspec.med.utoronto.ca/. [file 12915_2020_852_MOESM9_ESM.tif]

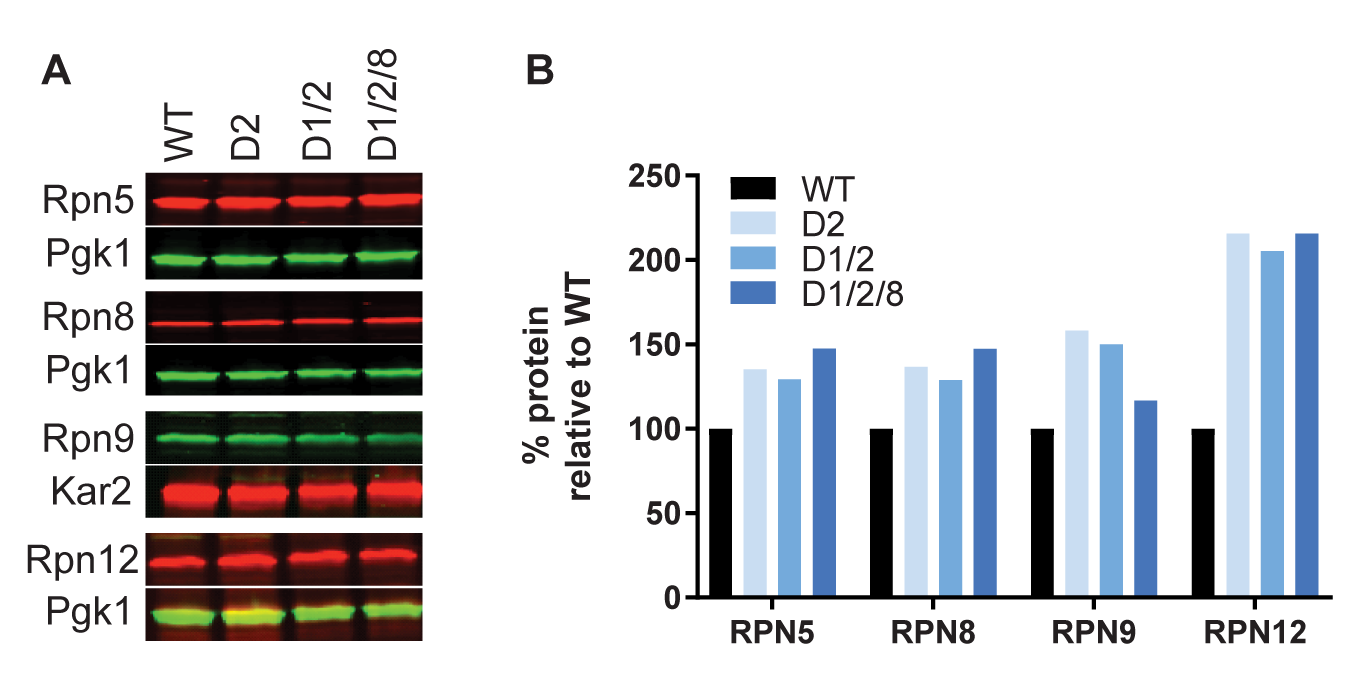

Supplement: Supplementary file 10 — Additional file 10: Figure S7. Aneuploid strains have elevated steady-state proteasomal subunit protein levels. (A) Steady-state levels of Rpn5, Rpn8, Rpn9, and Rpn12 proteins were measured by Western blot analysis in wild type (WT) and aneuploidy strains D2, D1/2 and D1/2/8. Proteins were resolved by SDS-PAGE and probed by immunoblotting using anti-Rpn5, anti-Rpn8, anti-Rpn9, or anti-Rpn12 antibodies. Endogenous Pgk1 or Kar2 were detected from the same membranes as a loading control using anti-Pgk1 or anti-Kar2 antibodies. (B) Quantification of indicated RPN protein levels based on the western blot shown in (A) with the relative protein levels normalized to the indicated loading control (one biological replicate performed for each strain and each respective probe). [file 12915_2020_852_MOESM10_ESM.tif]

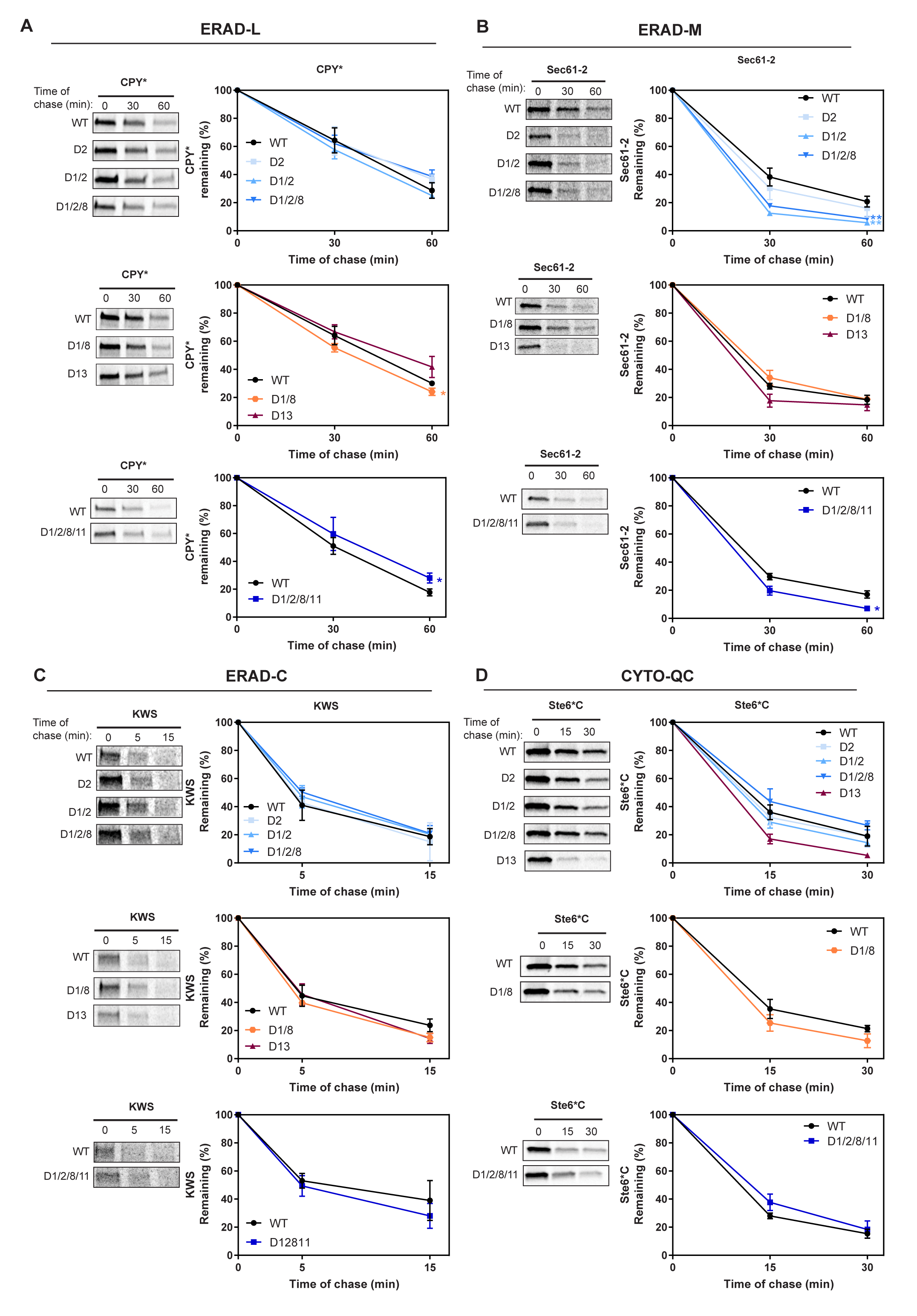

Supplement: Supplementary file 12 — Additional file 12: Figure S8. No defect in misfolded protein degradation capacity in aneuploid cells. A-D) Stability of misfolded substrate proteins in vivo subject to ER-associated degradation (ERAD) or cytosolic quality control (Cyto-QC) degradation pathways. Graphs corresponding to each batch (3 independent experiments per batch) are shown. Wild type (WT) and aneuploid strains were pulse-labeled for 10 min (CPY* and Sec61–2) or 5 min (KWS and Ste6*C) and chased for the times indicated. All proteins were immunoprecipitated using anti-HA antibodies and were resolved by SDS-PAGE and quantified using a phosphorimager. Representative phosphor screen scans are shown. Error bars represent the SD of three independent experiments. Turnover of (A) the ERAD-Luminal (ERAD-L) substrate CPY*, (B) ERAD-Membrane (ERAD-M) substrate Sec61–2, (C) ERAD-Cytosolic (ERAD-C) substrate KWS, (D) Cyto-QC substrate Ste6*C are shown. Student’s t test: *** p < 0.001, ** p < 0.01, * p < 0.05, and not significant p ≥ 0.05 is left unmarked. [file 12915_2020_852_MOESM12_ESM.tif]

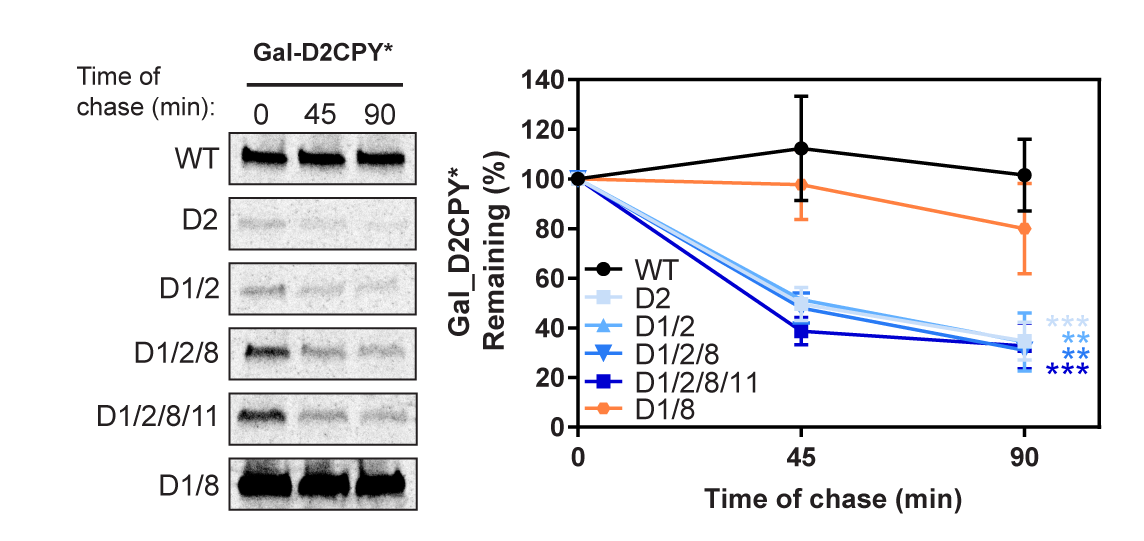

Supplement: Supplementary file 13 — Additional file 13: Figure S9. Enhanced degradation capacity in some aneuploid. Turnover of the degradation-deficient GAL-D2CPY* was monitored following pulse-labeling for 10 min as described previously in WT and aneuploid strains D2, D1/2, D1/2/8, D1/2/8/11 and D1/8 (data represent 3 independent experiments). Strains were grown overnight in Raffinose-containing media then induced for 4 h in galactose-containing media prior to chase for GAL-D2CPY* analysis. Student’s t test: *** p < 0.001, ** p < 0.01, * p < 0.05, and not significant p ≥ 0.05 is left unmarked. [file 12915_2020_852_MOESM13_ESM.tif]

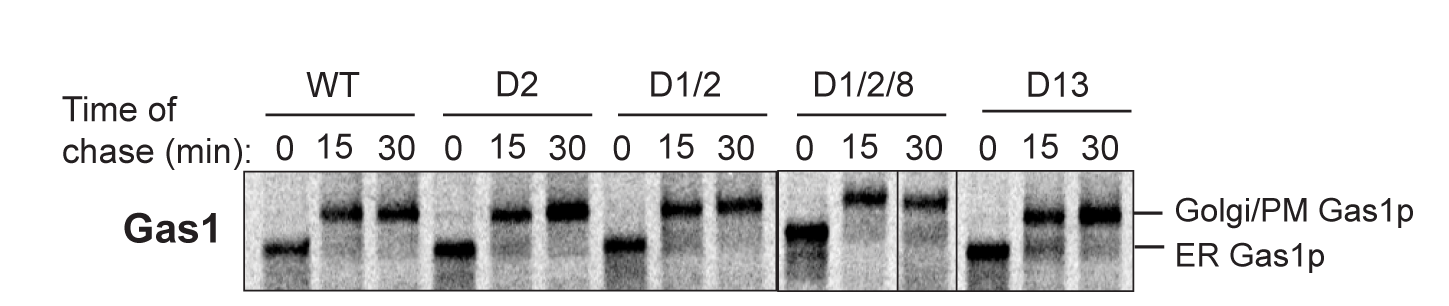

Supplement: Supplementary file 14 — Additional file 14: Figure S10. Aneuploid strains do not exhibit protein folding capacity defect or trafficking delay in aneuploid cells. Biosynthesis of the endogenous protein Gas1 was monitored following a 5 min pulse-label. Chase samples were taken at the time points indicated and Gas1 protein was immunoprecipitated using anti-Gas1 antibodies, followed by separation by SDS-PAGE and visualization and quantification by phosphorimager analysis. The ER (ER Gas1) and golgi/plasma membrane (Golgi/PM Gas1) forms are indicated in wild type (WT) and aneuploid strains D2, D1/2 and D1/2/8. Three independent experiments were performed, all yielding similar observations. Results from one representative experiment is shown. [file 12915_2020_852_MOESM14_ESM.tif]

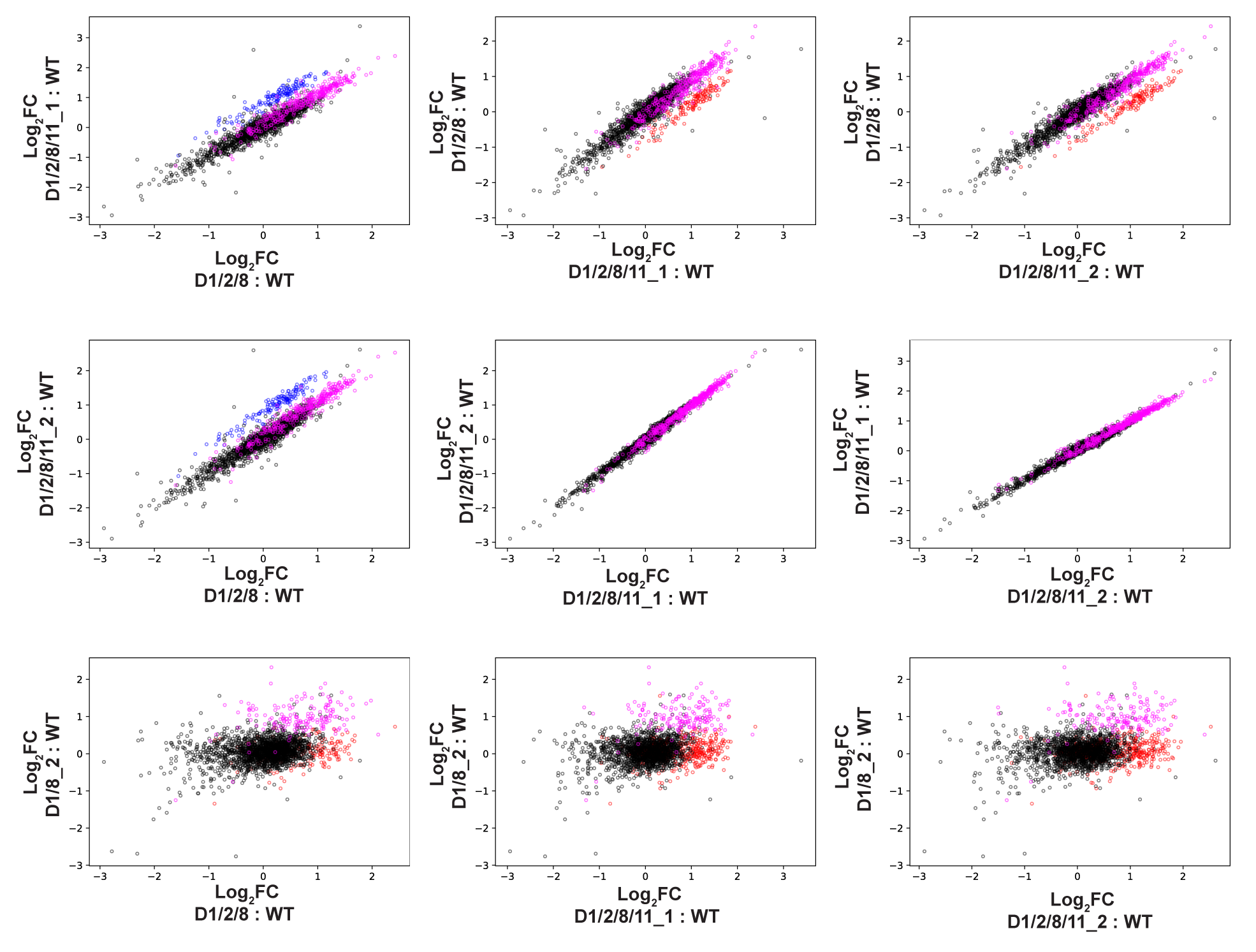

Supplement: Supplementary file 19 — Additional file 19: Figure S11. The mRNA log2 fold change of one strain relative to WT compared to another strain relative to WT. mRNA log2 fold changes in one strain compared to another strain, with each genes colored according to the chromosome in which it is located. Red dots are on a chromosome duplicated only in the x axis strain, blue dots are on a chromosome duplicated only on the y axis strain, purple dots are duplicated in both. [file 12915_2020_852_MOESM19_ESM.tif]
